# Bayesian mixed model analysis uncovered 21 risk loci for chronic kidney disease in boxer dogs

**DOI:** 10.1371/journal.pgen.1010599

**Published:** 2023-01-24

**Authors:** Frode Lingaas, Katarina Tengvall, Johan Høgset Jansen, Lena Pelander, Maria H. Hurst, Theo Meuwissen, Åsa Karlsson, Jennifer R. S. Meadows, Elisabeth Sundström, Stein Istre Thoresen, Ellen Frøysadal Arnet, Ole Albert Guttersrud, Marcin Kierczak, Marjo K. Hytönen, Hannes Lohi, Åke Hedhammar, Kerstin Lindblad-Toh, Chao Wang

**Affiliations:** 1 Faculty of Veterinary Medicine, Department of Preclinical Sciences and Pathology, Norwegian University of Life Sciences, Ås, Norway; 2 Science for Life Laboratory, Department of Medical Biochemistry and Microbiology, Uppsala University, Uppsala, Sweden; 3 Department of Clinical Sciences, Swedish University of Agricultural Sciences, Uppsala, Sweden; 4 BioVet Veterinary Laboratory AB, Sollentuna, Sweden; 5 Faculty of Biosciences, Norwegian University of Life Sciences, Ås, Norway; 6 Department of Cell and Molecular Biology, National Bioinformatics Infrastructure Sweden, Science for Life Laboratory, Uppsala University, Uppsala, Sweden; 7 Department of Medical and Clinical Genetics, University of Helsinki, Helsinki, Finland; 8 Department of Veterinary Biosciences, University of Helsinki, Helsinki, Finland; 9 Folkhälsan Research Center, Helsinki, Finland; 10 Broad Institute of MIT and Harvard, Cambridge, Massachusetts, United States of America; Clemson University, UNITED STATES

## Abstract

Chronic kidney disease (CKD) affects 10% of the human population, with only a small fraction genetically defined. CKD is also common in dogs and has been diagnosed in nearly all breeds, but its genetic basis remains unclear. Here, we performed a Bayesian mixed model genome-wide association analysis for canine CKD in a boxer population of 117 canine cases and 137 controls, and identified 21 genetic regions associated with the disease. At the top markers from each CKD region, the cases carried an average of 20.2 risk alleles, significantly higher than controls (15.6 risk alleles). An ANOVA test showed that the 21 CKD regions together explained 57% of CKD phenotypic variation in the population. Based on whole genome sequencing data of 20 boxers, we identified 5,206 variants in LD with the top 50 BayesR markers. Following comparative analysis with human regulatory data, 17 putative regulatory variants were identified and tested with electrophoretic mobility shift assays. In total four variants, three intronic variants from the *MAGI2* and *GALNT18* genes, and one variant in an intergenic region on chr28, showed alternative binding ability for the risk and protective alleles in kidney cell lines. Many genes from the 21 CKD regions, *RELN*, *MAGI2*, *FGFR2* and others, have been implicated in human kidney development or disease. The results from this study provide new information that may enlighten the etiology of CKD in both dogs and humans.

## Author summary

Chronic kidney disease (CKD) is described as a set of heterogeneous disorders affecting kidney structure and function. CKD is common in dogs and has been diagnosed in nearly all breeds. In this study, we identified 21 genetic regions associated with CKD in a boxer population and investigated the relevant genes and putative regulatory variants in these regions. Studies of canine CKD may help to better understand the pathology of kidney disease in both dogs and humans, and shows an important potential for early identification of high-risk individuals.

## Introduction

Chronic kidney disease (CKD) in humans is comprised of heterogeneous disease pathways that result in structural damage or decreased function presented as reduced glomerular filtration rate (GFR) or other markers of kidney disease for a period of more than three months [1].

In humans, CKD consists of clinically distinct disorders, also including congenital anomalies of both the kidney and urinary tract (CAKUT) and kidney disease secondary to hypertension and diabetes. This group of diseases represents a heavy and increasing disease burden in humans with prevalence estimates >10% and with substantial variation between human populations [2,3].

CKD also commonly occurs in dogs and cats [4,5]. The incidence of CKD in dogs is most likely between 0.5–1.5% and estimates from clinical data indicate that 10% of dogs over the age of 15 years are diagnosed with CKD [4]. Epidemiological analysis based on insurance data has shown an average incidence of kidney disease in general (without distinction between acute and chronic forms) of around 1.6% and significant differences in risk between Swedish dog breeds, with the highest incidence in the Bernese mountain dog, miniature schnauzer and boxer [5].

Although CKD may be initiated by environmental factors, a clear and significant genetic contribution has been shown in humans, strongly supported by heritability estimates of both disease and markers of kidney dysfunction, familial clustering, as well as linkage and genome-wide association studies (GWAS) [6–9]. It has become obvious that hundreds of genes contribute to complex forms of kidney disease as well as to the variation in markers associated with healthy kidneys [9,10]. Estimated glomerular filtration rate (eGFR), which is used as a marker for CKD in humans, was found to have high heritability (~29%) in a large biobank-study and more than 300 loci associated with eGFR-values were identified [11]. To date, more than 600 genes and loci have been implicated in monogenic and complex kidney diseases [12]. Causal variants associated with CKD may include rare monogenic or private variants, or common alleles with smaller effects. Additional variants are associated with kidney function markers, like eGFR and serum creatinine level [13].

A number of reports describe inherited renal disease in different dog breeds: Autosomal recessive hereditary nephropathy is noted in shih tzu [14], cocker spaniel [15], English springer spaniel [16], and Bernese mountain dog [17]; an autosomal dominant hereditary nephropathy in the bull terrier dogs [18]; and an X-linked hereditary nephropathy in Navasota dogs [19]. For decades, there have been concerns about a relatively high incidence of CKD in boxers. Based on pedigree studies breeders have suspected that CKD in young dogs commonly referred to as juvenile kidney disease (JKD), might have a genetic component. In a retrospective juvenile nephropathy study of 37 boxers less than five years old, Chandler[20] reported morphological findings of interstitial fibrosis, cell infiltration, dilated tubules and sclerotic glomeruli, Hoppe and Karlstam [21] described fetal, immature glomeruli and foci with small dysplastic structures surrounded by immature mesenchymal tissue in three CKD boxer puppies. Kolbjørnsen et al [22] studied morphological characteristics in seven related boxer dogs, three males and four females, between two months and five years. All cases had bilaterally small kidneys with segmental scarring and pronounced interstitial fibrosis. Across different studies of boxers, several dogs share these morphologic features of immature glomeruli, atypical tubules, proliferative arterioles and adenomatoid change [21,23,24]. Such features were less prominent in the study by Chandler [20], who also found a high frequency of incontinence and urinary tract infections in the studied boxers. Kolbjørnsen et al [22] classified the morphological features as reflux nephropathy or segmental hypoplasia. One case of juvenile nephropathy/nephronophthisis in boxers has also been reported [25].

The different studies show variation in morphology and time at onset, indicating that there may be a significant phenotypic and genetic heterogeneity also within the boxer breed. In this study, we aimed to uncover the genetic loci that contribute to canine CKD in a boxer population and explore candidate genes and functional variants within the associated regions. We hope this study will further improve our understanding of the genetic mechanism of CKD in dogs, which may in turn allow us to establish a canine model to facilitate the relevant studies in humans.

## Results

### CKD sample collection and genotyping

In this study, a total of 362 boxer samples were collected from Australia, Denmark, Finland, Germany, Norway, Sweden, UK and US (S1 Table). Only cases below six years of age and controls above eight years were included in this this study. The diagnosis of CKD in these dogs was supported by clinical characteristics, clinical pathology data, or a combination of them (see Materials and Methods). All individuals were genotyped with the Illumina Canine BeadChip. After removing samples for high-inbreeding, missingness, relatedness, and low supported phenotype, a final set of 117 cases and 137 controls with 101,664 markers was used for the association analysis, with UU_Cfam_GSD_1.0 (CanFam4) as the reference assembly.

### Candidate regions for CKD

**Fig 1:**
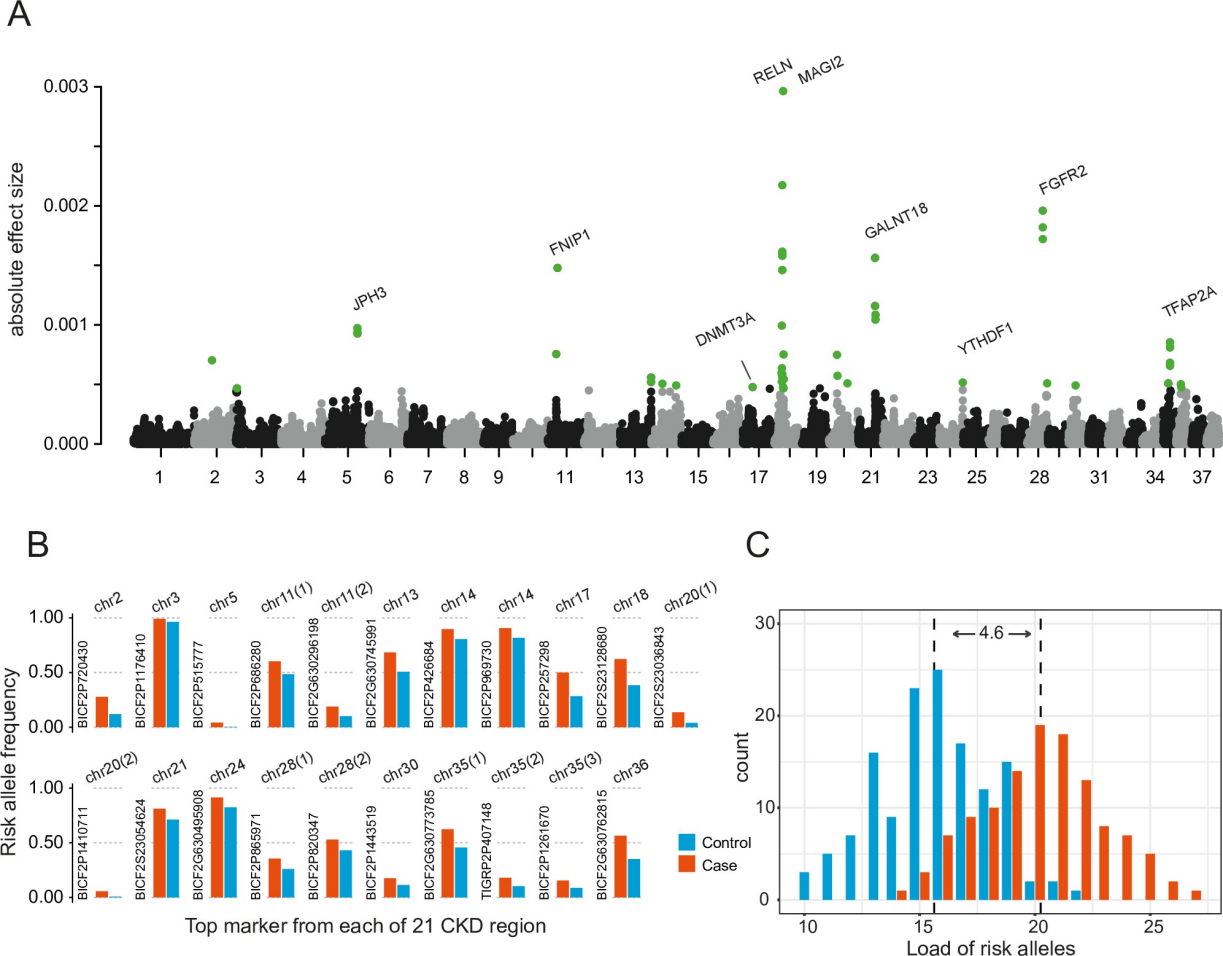
Association analysis of chronic kidney disease (CKD). (**A**) Manhattan plot of absolute SNP effect from BayesR analysis. The top 50 markers (green dots) with the highest effect were selected as candidates. 21 CKD regions were identified when merging linkage disequilibrium blocks of candidate markers. Genes relevant to kidney development or human disease were exhibited near the CKD region. (**B**) Risk allele frequency at the top marker from each of 21 CKD regions in cases (red) and controls (blue). Based on coordinates, appendix (1), (2), and (3) were added for the markers from the same chromosome. (**C**) Distribution of risk allele load for the cases (red) and controls (blue). On average, the cases carried 20.2 risk alleles, which is 4.6 higher than the controls (grey dash lines indicated the average load for cases and controls).

We estimated the effect size of markers for CKD using the BayesR algorithm. BayesR models the effect sizes of all variants simultaneously, provides unbiased estimates of the variants with larger effect sizes, with fewer false negatives and higher rate of true versus false positives, compared to traditional single-SNP GWAS with a linear mixed model [26]. The top 50 markers from Bayesian analysis were selected as disease associated candidates, presenting with an absolute effect size > 0.00047 (8.2 SDs from the mean; Fig 1A and S2 Table). We investigated these top 50 BayesR markers in 75 different dog breeds from a previous study [27], and the frequencies of the risk allele varied greatly among these breeds (S3 Table). Based on whole genome sequencing (WGS) data from 20 Norwegian boxers (S4 Table), we imputed genotypes on autosomes for all samples in the Bayesian set. A total of 5,206 imputed variants (3,924 SNPs + 1,282 Indels) were discovered in the strong linkage disequilibrium (LD, r^2^ > 0.9) with the Bayesian candidate markers and defined the CKD candidate regions.

Twenty-one CKD associated regions (Table 1 and S1 Fig) were detected across 15 autosomes with a total size of 15.9 Mb (mean region size of 756 Kb). At each CKD region, marker with the highest effect were selected, and their allele frequencies are illustrated in Fig 1B. The allele load for these 21 markers showed that the cases carried an average of 20.2 risk alleles, significantly higher than the 15.6 in the control group (Fig 1C, p< 2.2x10^-16^; t-test). With a cutoff of 17 risk alleles, the odds ratio of CKD status was 17.3 (95% CI: 8.5–35.2) between high and low load groups. This risk allele load was counted in the 75 other dog breeds mentioned above (S5 Table). In this boxer population, an ANOVA test indicated that these 21 top markers together explained 57% of phenotypic variation (S6 Table). A total of 146 protein coding genes were identified within or nearby (the closest gene on each side) the CKD regions (Table 1). Gene ontology analysis didn’t reveal enriched terms relevant to kidney development or disease. Interestingly, selection signals from dog domestication were found inside the two top CKD regions on chr18 and 28, and within a short distance from the CKD regions (< 300 Kb) on chr2, 11, and 14 (S7 Table).

**Table 1:** Candidate regions of chronic kidney disease from Bayesian analysis.

Chr	Start	End	Genes	Top marker	Risk/protective allele	Absolute effect size
chr18	13894632	19137370	*PRKAR2B*^*1*^, *ARMC10*, *ATXN7L1*, *CCDC146*, *CCDC71L*, *CDHR3*, *DNAJC2*, *EFCAB10*, *FAM185A*, *FBXL13*, *FGL2*, *GSAP*, *KMT2E*, *LHFPL3*, *LOC111090910*, *LOC111091026*, *LOC119877215*, *LOC119877227*, *LRRC17*, *MAGI2*, *NAMPT*, *NAPEPLD*, *ORC5*, *PHTF2*, *PIK3CG*, *PMPCB*, *PSMC2*, *PTPN12*, *PUS7*, *RELN*, *RINT1*, *RSBN1L*, *SLC26A5*, *SRPK2*, *SYPL1*, *TMEM60*, *GNAI1*^*2*^	BICF2S23128680	T/C	2.96E-03
chr28	30794656	31398277	*SEC23IP*^*1*^, *PLPP4*, *WDR11*, *FGFR2*^*2*^	BICF2P865971	C/G	1.96E-03
chr21	34721445	35231857	*LOC611563*^*1*^, *GALNT18*, *USP47*^*2*^	BICF2S23054624	T/C	1.56E-03
chr11	19343187	20249022	*CHSY3*^*1*^, *ACSL6*, *CDC42SE2*, *FNIP1*, *HINT1*, *LOC100685666*, *LOC608048*, *LYRM7*, *MEIKIN*, *RAPGEF6*, *IL3*^*2*^	BICF2G630296198	A/C	1.48E-03
chr5	65922431	66047923	*LOC119871920*^*1*^, *JPH3*, *KLHDC4*, *ZCCHC14*^*2*^	BICF2P515777	G/A	9.72E-04
chr35	15192697	15449129	*CD83*^*1*^, *JARID2*^*2*^	BICF2P1261670	T/C	8.52E-04
chr11	17505149	18064500	*FBN2*^*1*^, *ISOC1*, *SLC27A6*, *ADAMTS19*^*2*^	BICF2P686280	T/G	7.56E-04
chr20	16166851	16496650	*LOC119877302*^*1*^, *CNTN6*, *CHL1*^*2*^	BICF2S23036843	G/A	7.48E-04
chr2	36171795	36346340	*GNPDA1*^*1*^, *LOC111094597*, *NDFIP1*, *SPRY4*^*2*^	BICF2P720430	A/G	7.04E-04
chr35	14782605	14990410	*RNF182*^*1*^, *CD83*, *JARID2*^*2*^	TIGRP2P407148	T/C	6.81E-04
chr13	62465257	63189828	*NPFFR2*^*1*^, *ADAMTS3*, *ANKRD17*, *COX18*, *ALB*^*2*^	BICF2G630745991	T/C	5.59E-04
chr24	47293118	47757555	*GATA5*^*1*^, *COL9A3*, *DIDO1*, *GID8*, *LOC102155734*, *LOC119865717*, *LOC119877506*, *MRGBP*, *NTSR1*, *OGFR*, *SLC17A9*, *SLCO4A1*, *TCFL5*, *YTHDF1*^*2*^	BICF2G630495908	A/G	5.18E-04
chr28	40043492	40612284	*TCERG1L*^*1*^, *BNIP3*, *JAKMIP3*, *PPP2R2D*, *DPYSL4*^*2*^	BICF2P820347	G/A	5.09E-04
chr35	10883955	11451705	*LOC111093996*^*1*^, *LOC111093987*, *TFAP2A*^*2*^	BICF2G630773785	C/T	5.08E-04
chr20	36943764	36997397	*CACNA1D*^*1*^, *DCP1A*, *TKT*, *PRKCD*^*2*^	BICF2P1410711	C/T	5.08E-04
chr14	21555861	22230955	*SLC25A13*^*1*^, *DLX5*, *DLX6*, *SDHAF3*, *LOC100682772*^*2*^	BICF2P426684	C/T	5.06E-04
chr36	9385527	9637702	*FIGN*^*1*^, *GRB14*^*2*^	BICF2G630762815	C/T	5.02E-04
chr30	14100979	14537434	*LOC119877756*^*1*^, *LOC111093333*, *SEMA6D*, *SLC24A5*^*2*^	BICF2P1443519	T/C	4.92E-04
chr14	49731336	50705080	*DNAJB9*^*1*^, *IMMP2L*, *LRRN3*^*2*^	BICF2P969730	C/T	4.92E-04
chr17	18554108	19805628	*UBXN2A*^*1*^, *ADCY3*, *CENPO*, *DNAJC27*, *DNMT3A*, *DTNB*, *EFR3B*, *FAM228B*, *FKBP1B*, *ITSN2*, *LOC106559895*, *LOC119877151*, *LOC119877152*, *NCOA1*, *PFN4*, *POMC*, *PTRHD1*, *SF3B6*, *WDCP*, *ASXL2*^*2*^	BICF2P257298	A/G	4.78E-04
chr3	1096897	2091900	*NREP*^*1*^, *CAMK4*, *SLC25A46*, *STARD4*, *TMEM232*, *TSLP*, *WDR36*, *MAN2A1*^*2*^	BICF2P1176410	T/C	4.68E-04

1 the closest gene from the upstream of CKD region

2 the closest gene from the downstream of CKD region

### Functional annotation of variants in CKD regions

Using the UU_Cfam_GSD_1.0 annotation from NCBI, we investigated the genomic location of the 5,206 imputed variants. Most of them were present in intronic or intergenic regions (S8 Table). Fifteen variants occurred in coding regions, and four of these were nonsynonymous mutations, leading to amino acid changes in a total of 12 transcripts of four genes, *Membrane Associated Guanylate Kinase*, *WW And PDZ Domain Containing 2* (*MAGI2*), *Intersectin 2* (*ITSN2*), *Ankyrin Repeat Domain 17* (*ANKRD17*), and *Lysine Methyltransferase 2E* (*KMT2E*) genes (S9 Table). PROVEAN prediction on the effect suggested only the c.1784G>C (p.Arg595Thr, XM_038560916.1) in *ITSN2* as a deleterious mutation (PROVEAN score = -3.11) [28]. According to a canine variant dataset (Dog10K, 1929 individuals), this alternative allele G is rare in purebred dogs (F = 0.002), but common in the tested boxer population, for both cases (F = 0.51) and controls (F = 0.71). Considering its high frequency, we assumed the actual mutation effect could be relatively mild, otherwise it would be rapidly removed through breeding.

The large number of non-coding variants triggered our interest to investigate their potential regulatory function. We analyzed the phyloP constraint score for 3,924 imputed SNPs. 233 SNPs were found with intermediate score (phyloP > 1), of which 38 SNPs were highly constrained (phyloP >2.54, FDR < 5% in dog). The imputed SNPs were lifted to the human genome (hg38) to intersect with regulatory elements from three databases: Candidate cis-Regulatory Elements (cCREs, ENCODE) [29], promoter and enhancer (GeneHancer)[30], and DNase I hypersensitivity sites (HS, ENCODE)[30]. As a result, a total of 14 SNPs with phyloP > 2.54 were observed in cCRE, GeneHancer elements, or showed HS signals in more than 30 of 95 cell lines. Meanwhile, three additional SNPs with intermediate scores (phyloP between 1 to 2.56) were found in cCRE elements and showed strong HS signals in > 30 cell lines. With electrophoretic mobility shift assay (EMSA), we tested the regulatory function of these 17 putative regulatory SNPs (C1-C17; S10 Table and S2 Fig). Four SNPs, two from the introns of *MAGI2* (C8 and C9), one from the intron of *Polypeptide N-Acetylgalactosaminyltransferase 18* (*GALNT18*, C13), and one (C14) in the intergenic region on chr28, showed allele-specific binding in HEK293 or/and MDCK cell lines, implying the allele substitution at these positions could lead to change of protein-nucleic acid interaction.

### CKD region on chr18

**Fig 2:**
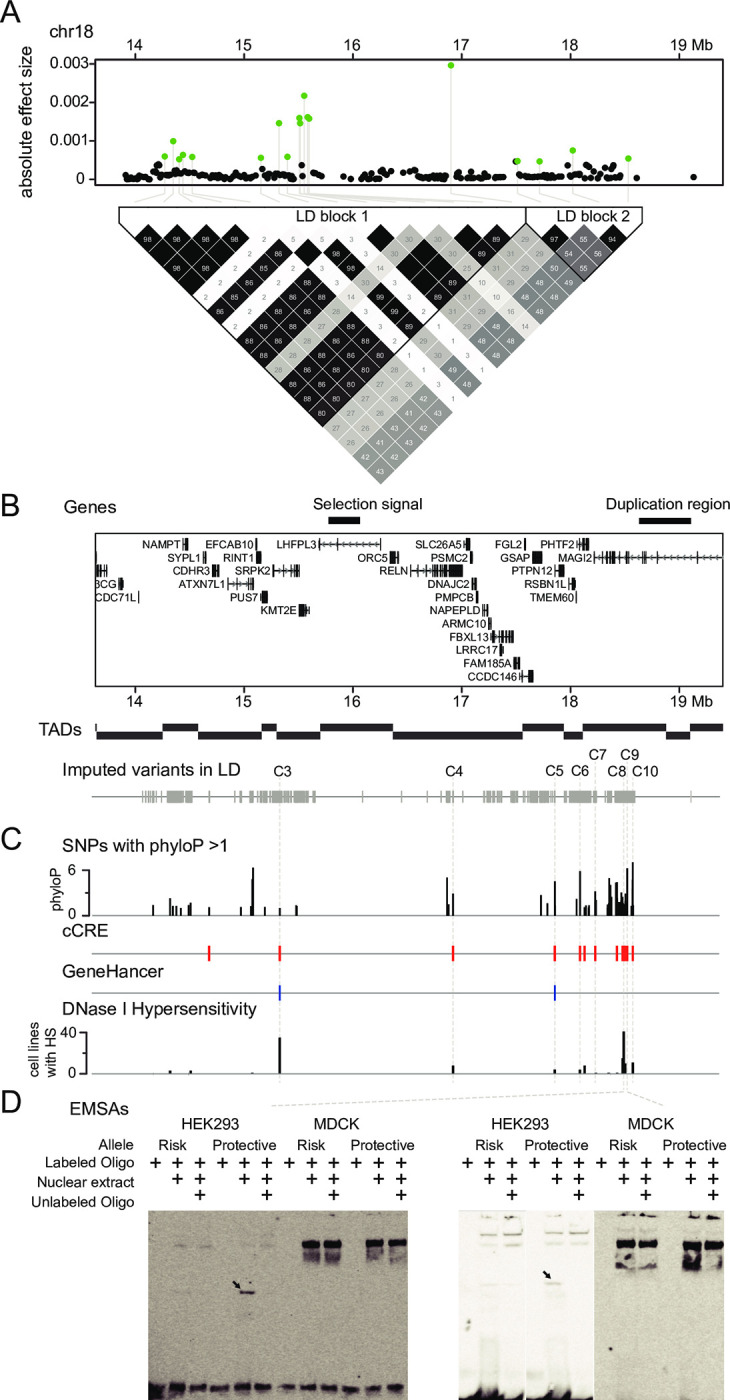
Chronic kidney disease (CKD) region on chr18 (chr18:13.9–19.1 Mb). (**A**) Eighteen Bayesian candidate markers (green dots) were observed in this region, and are present in two linkage disequilibrium (LD) blocks. (**B**) Annotated genes were identified around the CKD region. One selection signal of dog domestication and a ~500 Kb reported genomic duplicates were observed in this region. 2,064 imputed variants were found within the same LD of Bayesian candidate markers. (**C**) 76 imputed SNPs with phyloP score > 1 were lifted to human genome (hg38), and intersected with the regulatory elements from the candidate cis-Regulatory Elements (cCRE; red bars) and GeneHancer databases (blue bars), as well as the hypersensitivity (HS) signal from 95 cell lines (the height of the bar indicated the number of cell lines with the HS signal). (**D**) Eight putative regulatory SNPs (C3-C10) from the CKD region were tested with electrophoretic mobility shift assays (EMSAs). SNPs C8 and C9 showed alternative binding ability between the risk and protective alleles in HEK293 cell line.

The strongest Bayesian signal was found on chr18. A ~5.2 Mb CKD region consisted of two LD blocks and contained 18 Bayesian candidate markers (Fig 2A). The marker with the highest effect (BICF2S23128680; chr18: 16,900,760) was located in LD block 1, in the intron of the *Reelin* (*RELN*) gene (Fig 2B), and the risk allele frequency was 0.62 in cases and 0.38 in controls. *RELN* encodes a large extracellular glycoprotein that is required for specific biological activities at different times during embryonic development [31]. In the kidney, Reelin is highly expressed in proximal convoluted tubules and distal convoluted tubules in the early fetal stages of development, suggesting its participation in nephrogenesis [32]. Among the 17 putative regulatory SNPs mentioned above, eight of these reside in this CKD region (C3-C10; Fig 2C). The SNP C4 (chr18: 16,949,830) was identified from the intron of *RELN*, and was constrained among mammals (phyloP = 2.91) with a putative regulatory function (HS signals and cCRE enhancer; Fig 2C). However, EMSA analysis did not detect any protein-nucleic acid interaction on this site. Interestingly, metalloproteinase with thrombospondin motifs-3 (*ADAMTS3*), which encodes a protease that directly cleaves and inactivates reelin [33], was identified from the other CKD region on chr13. This implies that these two genes could contribute to CKD in the same pathway. Notably, the LD block 1 contained a selection signal (chr18:15.7–16.1 Mb), which was identified as the strongest signal in a demographically-based domestication study [34] and repeatedly reported in a parallel evolution analysis between dogs and humans [35].

From the other LD block 2, *MAGI2* is important for kidney barrier function [36]. Loss of *MAGI2* in podocytes can disrupt the slit diaphragm and morphologic abnormalities of foot processes in kidney [37]. In humans, MAGI2 is involved in the regulation of cytoskeletal rearrangement in podocytes, with its loss predisposing to proteinuria and CKD [38]. Within introns of the *MAGI2* gene, we identified four putative regulatory variants (C7-C10; Fig 2C), and two of these showed allele-specific binding in EMSAs (Fig 2D). The SNP C8 (chr18:18518972) had a relatively low phyloP score of 1.48, but was located in an enhancer element (EH38E2565734, cCRE) and overlapped with HS signals in 41 cell lines. EMSA exhibited much stronger binding for the C protective allele than the T risk allele in HEK293. According to the Dog10K dataset, the risk allele is rare in wolves (F = 0.07), but has a high frequency in both purebred dogs (F = 0.74) and village dogs (F = 0.51), indicating that the risk allele may be under selection during breeding or domestication. The SNP C9 sits at a highly constrained position (phyloP = 6.2) in an enhancer (EH38E2565751, cCRE). The EMSA binding band was strong for the G protective allele in HEK293 (Fig 2D).

### CKD region on chr28

**Fig 3:**
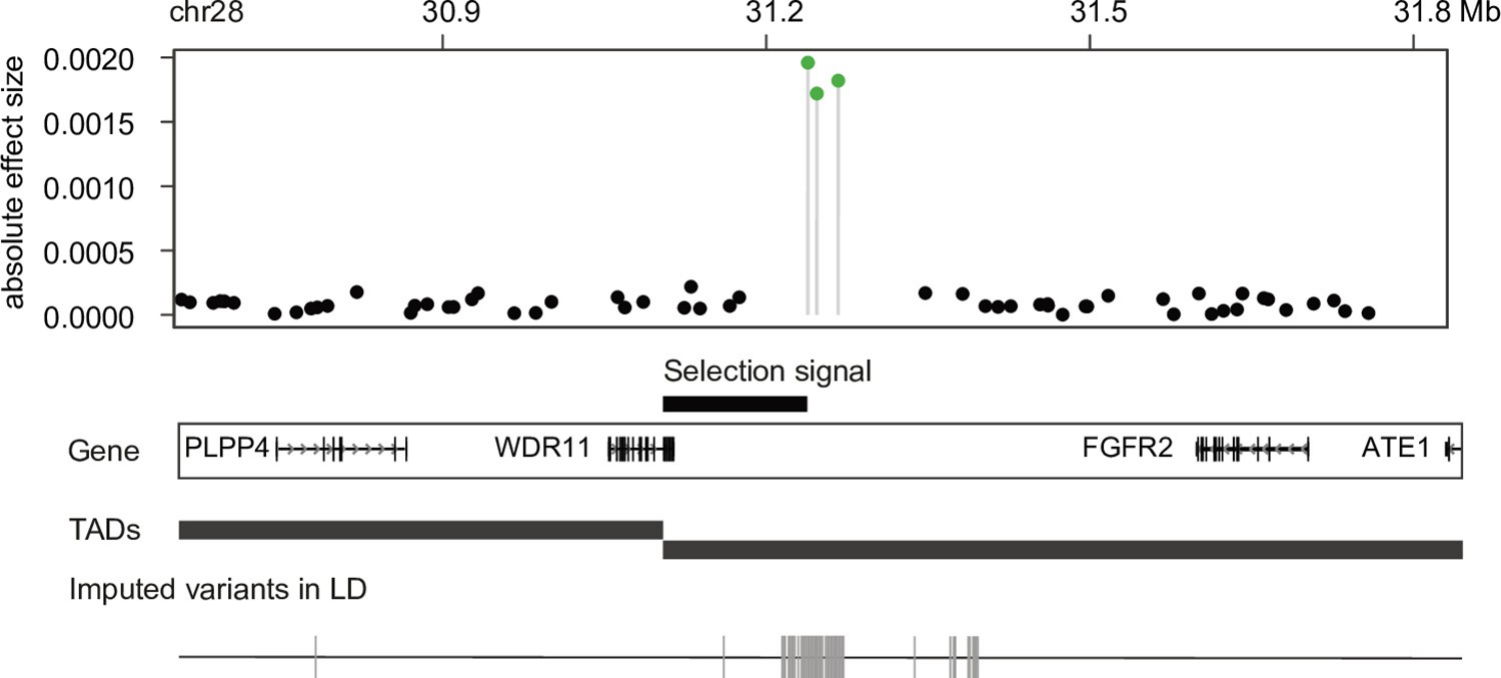
Chronic kidney disease (CKD) region on chr28 (chr28:30.8–31.4 Mb). Three candidate markers (green dots) from Bayesian analysis were found in this CKD region with the highest effect of 0.002. One selection signal was found nearby the candidate markers. Most of the imputed variants were located in the intergenic region between the *WDR11* and *FGFR2* genes in the same TAD.

The second strongest association was found in a region at chr28: 30.8–31.4 Mb (Fig 3), with the highest marker effect of 0.002 (BICF2P865971). Most imputed variants in LD (r^2^ > 0.9) were observed in the intergenic region between *WD Repeat Domain 11* (*WDR11*) and *Fibroblast growth factor receptor 2* (*FGFR2*). Mutations in *WDR11* can cause congenital hypogonadotropic hypogonadism (CHH) and Kallmann syndrome (KS) in humans [39], with unilateral or bilateral renal agenesis being a common phenotype in these patients [40]. FGFR2 is critical for early metanephric mesenchyme and ureteric bud formation in kidney [41]. *FGFR2* is 197 Kb outside of the CKD region, but resides in the same TAD domain as the top BayesR markers. There is a human *FGFR2* enhancer (GH10J121157) annotated in this CKD region, but no imputed variants were detected there. Interestingly, a reported domestication selection signal (chr28:31.1–31.2 Mb) was also found inside the CKD region.

### CKD region on chr21

**Fig 4:**
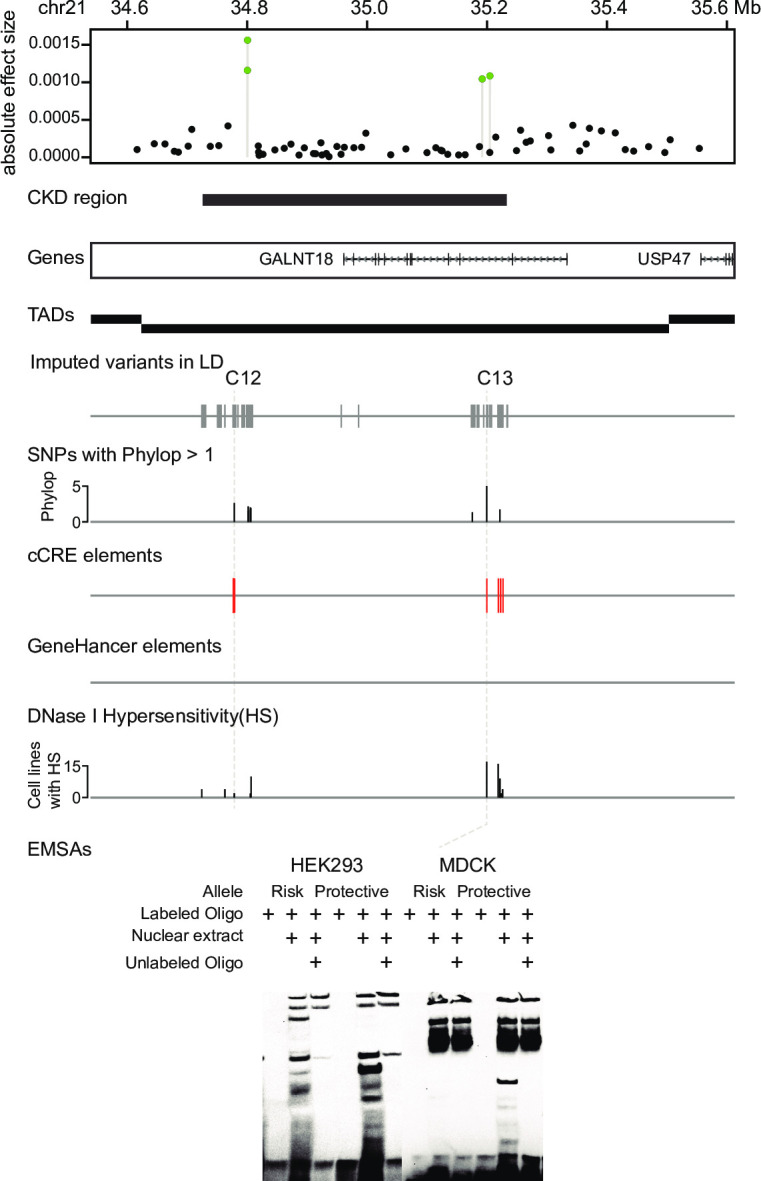
Chronic kidney disease (CKD) region on chr21 (chr21:34.7–35.2 Mb). Four Bayesian candidate markers (green dots) were identified in this 510 Kb CKD region. Within the LD of the candidate markers, two putative regulatory SNPs, C12 and C13, were identified from the downstream region and intron of *GALNT18* gene. EMSA validation illustrated allele-specific protein-nucleic acid binding of C13 in both HEK293 and MDCK cell lines.

A 510 Kb region on chr21 exhibited the third strongest association with CKD and includes four Bayesian candidate markers, with the highest effect of 0.0016 (BICF2S23054624; Fig 4). This region harbors only one gene, *GALNT18*, which is expressed ubiquitously in various human tissues including kidney [42]. A longitudinal analysis in lupus patients showed that the demethylation of *GALNT18* was associated with the development of active lupus nephritis [43]. Examination of imputed variants in the region revealed two putative regulatory SNPs, C12 and C13 (Fig 4). C12 is present at a constrained position (phyloP = 2.65) in an enhancer element (EH38E1520515; cCRE), 194 Kb downstream of *GALNT18*. C13 (phyloP = 5.05) is located within an intron of *GALNT18*, and overlaps with an enhancer element (EH38E1520796; cCRE), which showed HS signals in 17 cell lines. EMSA analysis revealed allele-specific binding of C13 in both HEK293 and MDCK alleles. The risk allele T is rare in wolves (F = 0.009), but had a 16 times higher frequency in purebred dogs (F = 0.14).

### Other CKD regions

Eighteen other CKD regions were identified on 14 autosomes, with the top SNP effects ranging from 0.00046 to 0.0015. By screening these CKD regions, we identified and functionally tested seven putative regulatory SNPs. From the CKD region at chr28:40–40.6 Mb, one putative regulatory SNP (C14) showed the allele-specific binding in both HEK293 and MDCK (S3A and S3B Fig). C14 is located at constrained position (phyloP = 4.3) of an enhancer element (EH38E1511661; cCRE) in an intergenic region. From the same LD block of C14, *BCL2-Interacting Protein 3* (*BNIP3*) plays a protective role against ischemia-reperfusion injury in renal tubular cells via regulating mitophagy [44].

## Discussions

In this study, we used a Bayesian approach and identified 21 genetic regions associated with CKD in boxer dogs. These loci together explain 57% of the phenotypic variation. Meanwhile, we screened variants in LD with the top 50 BayesR markers, and identified 17 putative regulatory SNPs in evolutionary constrained positions. EMSAs confirmed that four SNPs, from the introns of *MAGI2* and *GALNT18*, and an intergenic region of CKD on chr28, exhibited allele-specific binding in kidney cell lines, implying their potential function on CKD in boxers.

The process of domestication may have led to an increase in number and frequency of deleterious genetic variants, due to the artificial selection and population drift [45]. In this study, five CKD regions were identified around domestication selection signals. Additionally, dramatic changes in allele frequency between wolves and purebred dogs were found in two putative regulatory SNPs (C8 and C12). This suggests that selection or drift may be partially responsible for an increased prevalence of CKD in some dog breeds, and may also provide an insight into the origin of the genetic basis of disease.

Estimated LD extends ~50-fold greater distances within dog breeds than in humans [46]. In this study, the large region of 5.2 Mb on chr18 showed the strongest association with CKD. Although CKD candidate genes, like *RELN* and *MAGI2*, were easily recognized for their relevance to kidney function, it remains unclear if other genes from the locus or the interaction between them participate in CKD pathogenesis. To address the importance of these genes, it would be helpful to examine the CKD region in different breeds. Within the same TAD as the CKD region on chr28, the *FGFR2* is an interesting gene due to its function in the development of early embryos [47]. Two major splice variants of *FGFR2*, *FGFR2IIIb*, *and FGFR2IIIc*, were predominantly expressed in distinct tissues with differential ligand affinity [48]. *Fgfr2IIIb* null mice showed reduction in both kidney size and number of presumptive nephrons [49]. In our study, screening of human-based annotated elements did not reveal any potential regulatory variant in this gene. Thus, investigation of the dog-specific regulatory elements in the region may be helpful.

In addition to the top CKD regions, genes from other CKD regions may also be essential to kidney function. For example, near the CKD region on chr35 (chr35:10.8–11.4 Mb), transcription factor AP-2 alpha (TFAP2A) acts as a gatekeeper of differentiation during kidney development, by activating the terminal differentiation program of distal segments in the pronephros [50]. DNA (cytosine-5)-methyltransferase 3A (DNMT3A) from the CKD region on chr17 (chr17:18.5–19.8 Mb) is responsible for the methylation of gene regulatory regions that act as enhancers during kidney development [51]. Located in the CKD region on chr5 (chr5:65.9–66.0 Mb), *Junctophilin 3 (JPH3)* was identified in an association study of human CKD in 4,829 Japanese individuals [52]. *YTH N6-Methyladenosine RNA Binding Protein 1* (*YTHDF1*) near the CKD region on chr24 (chr24:47.2–47.7 Mb) was highly expressed in the human fibrotic kidneys as a key contributor for renal fibrosis [53]. *Folliculin interacting proteins-1* (*FNIP1*) was located in the CKD region on chr11 (chr11:19.3–20.2Mb). Disruption of FNIP1 resulted in the enlarged kidney size and significantly increased renal cyst formation [54].

At the 21 identified CKD loci, the load of risk alleles was significantly different between cases and controls (Fig 1C). Meanwhile, we investigated the risk alleles load of 21 CKD loci in 75 other breeds (S5 Table). It showed some breeds with high prevalence of CKD have a high-risk allele load, e.g., Cavalier King Charles Spaniel (22 alleles) [55], Collie (22 alleles), flat coated retriever (22 alleles) and Shih tzu (20 alleles) [5]. But other high-prevalence breeds were observed with a relatively low load, e.g. miniature schnauzer (18 alleles), boxer (17 alleles) [5] and Labrador retriever (17 alleles)[56]. This finding may indicate genetic heterogeneity, and that different sets of risk loci may play roles in CKD in other breeds. Therefore, the association of 21 risk loci and CKD in other breeds needs to be verified. To fully understand the genetics of CKD in canine, accurate diagnosis and sample collection from a wide range of breeds is required. This will aid cross-breed investigation, but will also increase the power to detect the loci with low effect through meta-analysis, and allow for fine mapping of shared CKD regions across breeds [46].

This analysis also has a potential value in risk estimation. In humans, risk scores based on the high number of identified CKD loci, such as polygenic risk scores (PRS)[57] and genomic risk score (GRS)[58], have been established for early identification of individuals at risk. A similar score system could be developed for canine CKD. We tentatively used the SNP effect from the BayesR analysis to estimate polygenic risk scores on 107 additional dogs, which were excluded from our original material due to the quality control. For this additional cohort predictions of polygenic risk scores yielded an odds ratio of 13.5 for being cases versus controls using risk scores below or above 0 as the decision threshold, where 0 was the average risk score. This finding will open important possibilities for early intervention and preventive measures for young dogs, and additionally provides a unique opportunity for selection of the breeding dogs at the lowest risk to reduce disease incidence. In conclusion, studies of canine CKD may help us understand the pathology of kidney disease in both dogs and human patients, and show an important potential of early identification of patients in predictive medicine.

## Materials and methods

### Ethics statement

All examination and sample collection of involved dogs were performed as part of the necessary diagnostic work-out by certified veterinarians according to ethical guidelines of the Norwegian University of Life Sciences or Swedish University of Agricultural Sciences. Sample collection in Finland was ethically approved by the Animal Ethics Committee of State Provincial Office of Southern Finland (ESAVI/343/04.10.07/2016). Written or verbal consents were obtained from the owners.

### CKD diagnosis

There is significant age variation in boxers affected by CKD. Still, because of the high prevalence of CKD in younger dogs, only cases below 6 years of age (average 2.6 years) were included in this study. The support for the diagnosis varied between samples and countries for both cases and controls and was based on a combination of available information and age. The diagnostic support was based on either i) clinical characteristics only. Evaluation of samples includes general clinical evaluation, with urine analysis, clinical chemistry with elevated creatinine, urea and/or SMDA ++; ii) clinics with clinical chemistry. Same as above, and with additional clinic chemistry test of serum sample at Norwegian University of Life Science, or iii) morphology evaluation with clinical data or clinical pathology data (S11 Table). Most samples with kidney tissues available were evaluated pathomorphologically in Norway or Sweden. Controls were collected from healthy elderly dogs (>8 years; average 9,8 years), with no known history of renal failure or urinary tract infections, and often supported with serum biochemistry analyses within reference intervals. For some control dogs, we were able to confirm a healthy kidney by morphology in older dogs euthanized for other reasons than kidney disease.

### Macroscopic renal lesions

**Fig 5:**
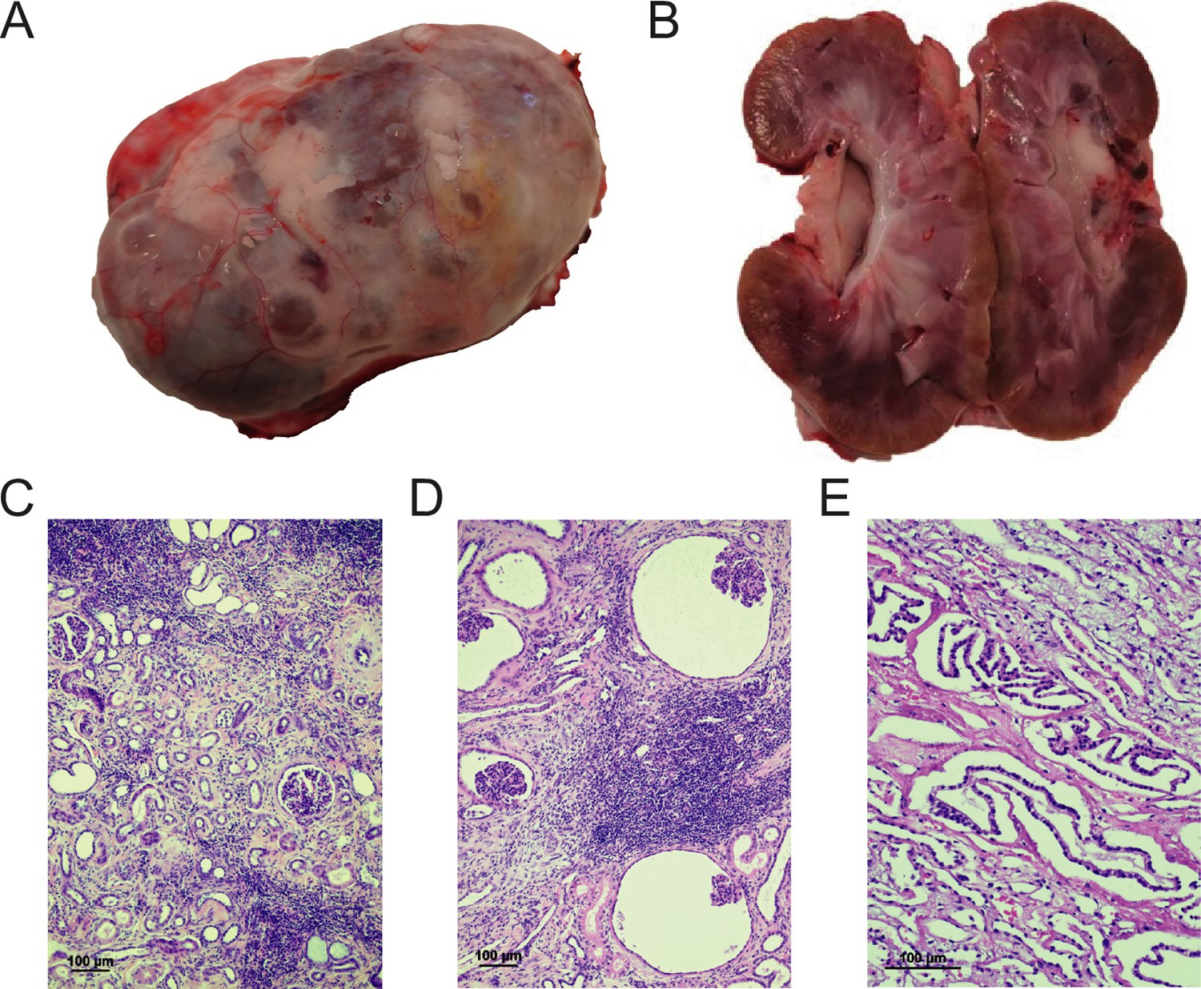
Pictures of boxer kidney with chronic kidney disease (CKD). (**A**) A non-decapsulated kidney from an 8-month-old female boxer dog with CKD revealing a coarse nodular irregular cortical surface; (**B**) sagittal section of the kidney revealing an irregularly pale and thinned cortex; (**C**) LM revealing cortical interstitial fibrosis with tubular atrophy and multifocal infiltration of mononuclear inflammatory cells, (**D**) glomerulocystic atrophy (HE x10) and (**E**) hyperplasia of medullary collecting duct epithelium consistent with so-called “atypical tubuli” or “adenomatoid change” (HE x20).

The typical cases showed bilaterally small, firm, and pale mottled kidneys with irregular surfaces. The cortical surfaces revealed coarse nodular irregularities with numerous segmental fibrotic depressions surrounded by nodular hypertrophic cortical tissue. The renal capsules were frequently adherent to the renal cortical surfaces. On the cut surfaces, the cortices were irregularly thinned with multifocal to coalescing pale radial scars causing depressions of the cortical surfaces (Fig 5A and 5B).

### Histological renal lesions

The fibrotic segments of the renal cortex were characterized by variable and often multifocal infiltration of mononuclear inflammatory cells (lymphocytes and plasma cells) and tubular atrophy and paucity of glomeruli combined with enlargement of the interstitial connective tissue (Fig 5C). Tubular microcysts and glomerulocystic atrophy were observed within the fibrotic segments (Fig 5D). Medullary fibrosis and variable multifocal lymphoplasmacytic infiltration were common findings. Both in the juxtamedullary cortex and in the medulla epithelial hyperplasia of collecting ducts was frequently giving the impression of “atypical tubules” also called “adenomatoid tubular change” (Fig 5E). Glomeruli in the surrounding hypertrophic cortex often revealed varying segmental or global sclerosis. Fibrotic thickening around and along the Bowman capsules was a frequent finding.

### DNA extraction and genotyping

Blood samples were collected for 362 boxers from Australia, Denmark, Finland, Germany, Norway, Sweden, UK and US (S1 and S12 Tables). DNA mini kit (QIAGEN, Germany) was used for DNA extraction from the blood sample with standard protocol. DNA was quantified by Nanodrop 2000 and stored at -20°C. The genotyping was performed at the SNP & SEQ Technology Platform in Uppsala or GeneSeek (Neogen, US) using Illumina CanineHD BeadChip.

### Quality control

Of the 362 genotyped samples, we excluded one duplicate sample, and dogs with missing phenotype record (one dog), failed to meet the diagnosis criteria (14 dogs), or age criteria (three dogs). Six samples were identified and removed as outliers in inbreeding analysis with plink (v 1.90b4.9) [59], and 22 dogs were filtered out due to an overall call rate < 90%. The relatedness was tested by the plink2 with KING [60] kingship cutoff of 0.15, and 61 dogs were removed at this step. UU_Cfam_GSD_1.0 (CanFam4; NCBI assembly: GCA_011100685.1) was used reference assembly in this study [61]. Markers with genotyping rate < 95% (55,597 markers) and minor allele frequency < 1% (47,668 markers) were excluded. A final dataset consisting of 254 boxers with 101,664 autosomal markers were used for the analysis.

### Population structure

We performed principal components analysis (PCA) with autosomal markers to evaluate population structure with plink (v 1.90b4.9) [59]. The first two components explained 15.5% and 6.5% genetic variation respectively. The PCA plot illustrated that boxers with different country of origin were mixed in one cluster (S4 Fig). Notably, the majority of boxers from the US (25 of 27) were slightly distanced from the others. However, since the controls and cases were evenly distributed across the population with no obvious stratification, all boxers were considered as one single group. We conducted a variance component analysis using WOMBAT (v 17/04/2014) [60], which showed a genomic relationship matrix (GRM)-based heritability of CKD was 0.61 ± 0.09 in this population.

### Bayesian association analysis

We used the BayesR (v 01/04/2021) algorithm [26] to perform a genome-wide association analysis of CKD in the boxer population. BayesR assumes that the SNP effects are a priori derived from a mixture of four normal distributions: N(0, 0), N(0, 0.0001 $\sigma_{g}^{2}$), N(0, 0.001 $\sigma_{g}^{2}$) or N(0, 0.01 $\sigma_{g}^{2}$). SNP effects from the four distributions were estimated using the Markov Chain Monte Carlo (MCMC) sampling. BayesR was run with the first principal component as the covariate for a total of 300,000 iterations with a burn-in step of 100,000. The model convergence was assessed from ten BayesR repeats runs, and top 50 markers with the highest absolute effect size were considered as the candidates, according to a previous study [62]. The frequency of these top 50 markers were investigated in other 75 breeds (sample size > = 10) from a previous study [27]. LD block analysis of candidate BayesR markers was performed and visualized with Haploview (v 4.2) [63].

### Genome sequencing and variant calling

To discover common variants from the candidate regions, we generated WGS data for 20 Norwegian boxers (12 cases and eight controls, S4 Table). Illumina short reads libraries preparation and sequencing were performed by the Norwegian Sequencing Centre at University of Oslo in Norway. The paired-end reads were mapped to dog UU_Cfam_GSD_1.0 reference [61] using BWA-mem2 (v 2.1) [64]. The alignment was sorted and indexed by SAMtools (v 1.14) [65]. Duplicate reads were detected and marked by the MarkDuplicates module of Picard Toolkit (v 2.27.5; Broad Institute).

The SNPs and INDELs were called using the HaplotypeCaller from GATK (v 4.2.0.0) [66]. Afterward, a joint genotyping analysis was performed using CombineGVCFs and GenotypeGVCFs, to merge variants for samples in one cohort. Only biallelic SNPs and indels were selected and filtered using SelectVariants, with “hard-filtering” parameters “QD < 2.0 || FS > 60.0 || MQ < 40.0 || MQRankSum < −12.5 || ReadPosRankSum < −8.0” and “QD < 2.0 || FS > 200.0 || ReadPosRankSum < −20.0”, respectively.

### Genotype imputation and validation

The WGS genotypes from 20 boxers were phased using SHAPEIT2 (v 2.r904) [67], to generate a reference pool of haplotypes. SNP chip data were pre-phased with SHAPEIT2. Genotype imputation was performed using IMPUTE2 (v 2.3.2) [68] by comparing the chip data haplotype with the reference pool. Imputed genotypes with probability > 0.9 were kept, otherwise they were set as missing. Specifically, a ~500 Kb region on chr18 was masked during the imputation, which is known as a genomic duplication caused by the orthologous segments on chr9 [61]. The imputed variants were filtered by requiring a minor allele frequency > 0.01, and call rate > 0.95.

Internal cross-validation from IMPUTE2 indicated an imputation concordance of 97.7%. Meanwhile, we designed the primers and validated two imputed variants using sanger sequencing: 11bp indel (chr18: 16811242; forward: TCCCAAGCCAAACTCTGTTC; reverse: CCTGAAATGGCCTCTTTCTC) and one T/C SNP (chr18: 16914812; forward: ATTTGTCCCTGGCATTCTTG; reverse: GGGTCTCATTAGGCCCTGTT), which showed concordances of 100% (135/135) and 98.8% (168/170) respectively.

We further cross validated the imputation with seven boxers from previous studies [69–71]. The WGS data of seven samples were downloaded and mapped to the canfam4 with coverages ranging from 17-28x (S13 Table). For these boxers, the genotypes were called and filtered (GQ > 30) at 7,264,546 variants (5,601,532 SNPs and 1,663,014 Indels) that were identified in the reference panel of 20 Norwegian boxers (HaplotypeCaller in GATK). The imputation was performed based on a subset of genotypes at 104,839 markers from the illumina chip. Afterward, we validated the imputed genotypes by comparing to the genotypes called from WGS data, with–sample-diff function in plink2 (v alpha-2.3)[59], which revealed an average of imputation rate (successfully imputed; genotype probability > 0.9 in IMPUTE2) of 95%, and imputation accuracy of 96% (correctly imputed, S13 Table).

### CKD candidate regions

Imputed variants with high LD (r^2^>0.9) to any of the top 50 BayesR markers were detected with plink (-r2 -ld-window -r2 0.9 -ld-window-kb 1000 -ld-window 5000). LD blocks within 500 Kb were merged, and 21 candidate CKD regions were defined by variant position at the ends of LD block. The pairwise interaction of 21 BayesR markers were screened using “–fast-epistasis” function with BOOST method [72] implemented in plink, and no significant interaction was found. BayesR markers with the highest effect size were selected from each of 21 CKD regions. To assess the phenotypic variation explained by the identified CKD regions, we determined the association of phenotype and 21 BayesR markers with analysis of variance (ANOVA) in R (v 4.0.1; aov function) with model “phenotype ~ SNP1+SNP2+…+SNP21”. The means of risk alleles load between control and case groups were counted and compared with t-test in R (ttest function). Additionally, we counted the risk allele load individually, then calculated the average load for the 75 breeds described in the Bayesian association analysis.

### Gene annotation and gene ontology analysis

Protein-coding genes within the CKD regions and the closest gene on each side were identified based on UU_Cfam_GSD_1.0 annotation from NCBI (GCF_011100685.1). Genes were assigned to the human orthologues for the gene ontology (GO) analysis with Metascape [73].

### Overlap of candidate variants with genomic features

To investigate if dog domestication contributes to CKD, we compared CKD regions with selection signals from four studies [34,35,74,75]. Imputed variants in LD with BayesR markers were evaluated by comparing different annotated genomic features: 1) variant effect was annotated using SNPeff (v 4.3t)[76]; 2) location to topologically associating domains (TAD) in CKD regions were extracted from a previous study in dog liver tissue[77]; 3) risk allele frequency of imputed variants was extracted from Dog10K dataset (1,591 purebred dogs, 281 village dogs and 57 wolves; https://kiddlabshare.med.umich.edu/dog10K/) [69]; 4) The phyloP scores from 241 mammals were extracted from the Zoonomia project[78]. To obtain an extended functional annotation, imputed SNPs were lifted to the human hg38 genome using LiftOver (https://genome.ucsc.edu/cgi-bin/hgLiftOver); 5) the lifted SNPs were intersected with the human regulatory elements from cCREs and HS databases from ENCODE[29,79], and with the promoters and enhancers from GeneHancer [30].

### Electrophoretic mobility shift assay (EMSA)

Putative regulatory SNPs were selected and tested with EMSA in Madin-Darby Canine Kidney (MDCK) and human embryonic kidney (HEK293) cell lines. All cell lines were cultured in DMEM (Gibco), supplemented with 10% heat inactivated foetal bovine serum (Gibco), 1% penicillin, streptomycin and glutamine (Gibco) and maintained at 37°C (5% CO_2_). For EMSAs, nuclear extracts from each cell line were prepared according to the manufacturer’s specification (NucBuster Protein Extraction Kit, Merck) and assayed using the appropriate oligo set (S14 Table) and with the Lightshift Electrophoretic Mobility-Shift Assay kit (Thermo Fisher Scientific). The following alterations were made to the manufacturer’s protocol: 12–15 μg of appropriate nuclear extract was pre-incubated on ice for 40 minutes in binding buffer (binding buffer supplemented with: 7.5% Glycerol, 0.063% NP-40, 30.1 mM KCl, 2 mM MgCl2, 0.1 mM EDTA, 50 ng/ul Poly (dI·dC)). Biotin labelled ds-oligonucleotides were added at 200 fmol and competed where appropriate with matched 20 pmol unlabelled ds-oligonucleotides. Reactions were incubated on ice for 40 minutes prior resolution on a 5% polyacrylamide gel (BioRad) run in 0.5 × TBE at 100 V for one and half hours. For four variants which showed allele-specific binding in the EMSA, a technical replicate was performed to validate the result.

## Supporting information

S1 Table Origin of boxers used in this study. (DOCX)

S2 Table Top 50 markers from Bayesian analysis. (DOCX)

S3 Table Risk allele frequency of top 50 BayesR markers in 75 breeds. (XLSX)

S4 Table Summary of whole genome sequencing data of 20 Norwegian boxers. (DOCX)

S5 Table Average of risk allele load of 21 chronic kidney disease loci in 75 breeds. (DOCX)

S6 Table ANOVA test of 21 BayesR markers with the chronic kidney disease. (DOCX)

S7 Table Selection signals from domestication found around the chronic kidney disease regions. (DOCX)

S8 Table Location of 5,206 imputed variants in chronic kidney disease regions. (DOCX)

S9 Table Missense mutations detected in chronic kidney disease regions. (DOCX)

S10 Table Seventeen variants identified with putative regulatory function. (DOCX)

S11 Table Diagnosis of chronic kidney disease in boxers. (DOCX)

S12 Table Information of boxer samples used in this study. (XLSX)

S13 Table Cross validation of imputation with seven boxers. (DOCX)

S14 Table Primer sequences for the EMSA assays. (DOCX)

S1 Fig Absolute effect of top 50 BayesR markers from 15 autosomes. The top 50 markers (blue dots; corresponding to a threshold of 0.000467, grey dash line) from Bayesian association analysis were from 15 different autosomes (chromosome numbers were labeled on the right side of each panel).
(DOCX)

S2 Fig Flow chart of 17 putative regulatory SNPs selection. Imputed SNPs in LD with top markers from Bayesian analysis were compared with the candidate Cis-Regulatory Elements (cCRE, ENCODE), promotor and enhancer elements (GeneHancer), and Hypersensitivity (HS) signals in 95 human cell lines.
(DOCX)

S3 Fig Chronic kidney disease (CKD) region on chr28. (A) A 569 Kb CKD region was found on chr28. It contains one candidate marker (green dot) from Bayesian analysis. (B) A putative regulatory SNP (C14) was found in the intergenic region in the CKD region. EMSA confirmed the allele-specific binding of C14 in both HEK293 and MDCK cell lines.
(DOCX)

S4 Fig The principal component analysis (PCA) plot showed population structure of 254 boxers. The first two components explained 15.5% and 6.5% genetic variation, respectively. In general, boxers with different origins were mixed as one cluster, with controls and cases evenly distributed.
(DOCX)

## References

[1] Webster AC, Nagler EV, Morton RL, Masson P. Chronic Kidney Disease. Lancet Lond Engl. 2017;389: 1238–1252. doi: 10.1016/S0140-6736(16)32064-5 27887750

[2] Hill NR, Fatoba ST, Oke JL, Hirst JA, O’Callaghan CA, Lasserson DS, et al. Global Prevalence of Chronic Kidney Disease–A Systematic Review and Meta-Analysis. PLOS ONE. 2016;11: e0158765. doi: 10.1371/journal.pone.0158765 27383068 PMC4934905

[3] Bikbov B, Purcell CA, Levey AS, Smith M, Abdoli A, Abebe M, et al. Global, regional, and national burden of chronic kidney disease, 1990–2017: a systematic analysis for the Global Burden of Disease Study 2017. The Lancet. 2020;395: 709–733. doi: 10.1016/S0140-6736(20)30045-3 32061315 PMC7049905

[4] Bartges JW. Chronic Kidney Disease in Dogs and Cats. Vet Clin Small Anim Pract. 2012;42: 669–692. doi: 10.1016/j.cvsm.2012.04.008 22720808

[5] Pelander L, Ljungvall I, Egenvall A, Syme H, Elliott J, Häggström J. Incidence of and mortality from kidney disease in over 600,000 insured Swedish dogs. Vet Rec. 2015;176: 656. doi: 10.1136/vr.103059 25940343

[6] Wuttke M, Köttgen A. Insights into kidney diseases from genome-wide association studies. Nat Rev Nephrol. 2016;12: 549–562. doi: 10.1038/nrneph.2016.107 27477491

[7] Chambers JC, Zhang W, Lord GM, van der Harst P, Lawlor DA, Sehmi JS, et al. Genetic loci influencing kidney function and chronic kidney disease. Nat Genet. 2010;42: 373–375. doi: 10.1038/ng.566 20383145 PMC3748585

[8] Devuyst O. Genetic Variants and Risk of Chronic Kidney Disease. Perit Dial Int J Int Soc Perit Dial. 2014;34: 150. doi: 10.3747/pdi.2014.00063 24676739 PMC3968098

[9] Tin A, Köttgen A. Genome-Wide Association Studies of CKD and Related Traits. Clin J Am Soc Nephrol CJASN. 2020;15: 1643–1656. doi: 10.2215/CJN.00020120 32409295 PMC7646230

[10] Rasouly HM, Groopman EE, Heyman-Kantor R, Fasel DA, Mitrotti A, Westland R, et al. The Burden of Candidate Pathogenic Variants for Kidney and Genitourinary Disorders Emerging From Exome Sequencing. Ann Intern Med. 2019;170: 11–21. doi: 10.7326/M18-1241 30476936

[11] Wuttke M, Li Y, Li M, Sieber KB, Feitosa MF, Gorski M, et al. A catalog of genetic loci associated with kidney function from analyses of a million individuals. Nat Genet. 2019;51: 957–972. doi: 10.1038/s41588-019-0407-x 31152163 PMC6698888

[12] KDIGO Conference Participants. Genetics in chronic kidney disease: conclusions from a Kidney Disease: Improving Global Outcomes (KDIGO) Controversies Conference. Kidney Int. 2022;101: 1126–1141. doi: 10.1016/j.kint.2022.03.019 35460632 PMC9922534

[13] Groopman EE, Povysil G, Goldstein DB, Gharavi AG. Rare genetic causes of complex kidney and urological diseases. Nat Rev Nephrol. 2020;16: 641–656. doi: 10.1038/s41581-020-0325-2 32807983 PMC7772719

[14] Hoppe A, Swenson L, Jönsson L, Hedhammar A. Progressive nephropathy due to renal dysplasia in shih tzu dogs in Sweden: A clinical pathological and genetic study. J Small Anim Pract. 1990;31: 83–91. doi: 10.1111/j.1748-5827.1990.tb00728.x

[15] Davidson AG, Bell RJ, Lees GE, Kashtan CE, Davidson GS, Murphy KE. Genetic cause of autosomal recessive hereditary nephropathy in the English Cocker Spaniel. J Vet Intern Med. 2007;21: 394–401. doi: 10.1892/0891-6640(2007)21[394:gcoarh]2.0.co;2 17552442

[16] Nowend KL, Starr-Moss AN, Lees GE, Berridge BR, Clubb FJ, Kashtan CE, et al. Characterization of the genetic basis for autosomal recessive hereditary nephropathy in the English Springer Spaniel. J Vet Intern Med. 2012;26: 294–301. doi: 10.1111/j.1939-1676.2012.00888.x 22369189

[17] Minkus G, Breuer W, Wanke R, Reusch C, Leuterer G, Brem G, et al. Familial nephropathy in Bernese mountain dogs. Vet Pathol. 1994;31: 421–428. doi: 10.1177/030098589403100403 7941230

[18] Hood JC, Robinson WF, Huxtable CR, Bradley JS, Sutherland RJ, Thomas MA. Hereditary nephritis in the bull terrier: evidence for inheritance by an autosomal dominant gene. Vet Rec. 1990;126: 456–459. 2356601

[19] Benali SL, Lees GE, Nabity MB, Aricò A, Drigo M, Gallo E, et al. X-Linked Hereditary Nephropathy in Navasota Dogs: Clinical Pathology, Morphology, and Gene Expression During Disease Progression. Vet Pathol. 2016;53: 803–812. doi: 10.1177/0300985815624494 26917550

[20] Chandler ML, Elwood C, Murphy KF, Gajanayake I, Syme HM. Juvenile nephropathy in 37 boxer dogs. J Small Anim Pract. 2007;48: 690–694. doi: 10.1111/j.1748-5827.2007.00401.x 17727634

[21] Hoppe A, Karlstam E. Renal dysplasia in boxers and Finnish harriers. J Small Anim Pract. 2000;41: 422–426. doi: 10.1111/j.1748-5827.2000.tb03237.x 11023130

[22] Kolbjørnsen O, Heggelund M, Jansen JH. End-stage kidney disease probably due to reflux nephropathy with segmental hypoplasia (Ask-Upmark kidney) in young Boxer dogs in Norway. A retrospective study. Vet Pathol. 2008;45: 467–474. doi: 10.1354/vp.45-4-467 18587092

[23] Lucke VM, Kelly DF, Darke PG, Gaskell CJ. Chronic renal failure in young dogs—possible renal dysplasia. J Small Anim Pract. 1980;21: 169–181. doi: 10.1111/j.1748-5827.1980.tb01229.x 7366181

[24] Cavalera MA, Gernone F, Uva A, D’Ippolito P, Roura X, Zatelli A. Clinical and Histopathological Features of Renal Maldevelopment in Boxer Dogs: A Retrospective Case Series (1999–2018). Animals. 2021;11: 810. doi: 10.3390/ani11030810 33805804 PMC8001074

[25] Basile A, Onetti-Muda A, Giannakakis K, Faraggiana T, Aresu L. Juvenile nephropathy in a Boxer dog resembling the human nephronophthisis-medullary cystic kidney disease complex. J Vet Med Sci. 2011;73: 1669–1675. doi: 10.1292/jvms.10-0551 21836389

[26] Moser G, Lee SH, Hayes BJ, Goddard ME, Wray NR, Visscher PM. Simultaneous Discovery, Estimation and Prediction Analysis of Complex Traits Using a Bayesian Mixture Model. PLoS Genet. 2015;11: e1004969. doi: 10.1371/journal.pgen.1004969 25849665 PMC4388571

[27] Parker HG, Dreger DL, Rimbault M, Davis BW, Mullen AB, Carpintero-Ramirez G, et al. Genomic Analyses Reveal the Influence of Geographic Origin, Migration, and Hybridization on Modern Dog Breed Development. Cell Rep. 2017;19: 697–708. doi: 10.1016/j.celrep.2017.03.079 28445722 PMC5492993

[28] Choi Y, Chan AP. PROVEAN web server: a tool to predict the functional effect of amino acid substitutions and indels. Bioinforma Oxf Engl. 2015;31: 2745–2747. doi: 10.1093/bioinformatics/btv195 25851949 PMC4528627

[29] ENCODE Project Consortium Moore JE, Purcaro MJ Pratt HE, Epstein CB, Shoresh N, et al. Expanded encyclopaedias of DNA elements in the human and mouse genomes. Nature. 2020;583: 699–710. doi: 10.1038/s41586-020-2493-4 32728249 PMC7410828

[30] Fishilevich S, Nudel R, Rappaport N, Hadar R, Plaschkes I, Iny Stein T, et al. GeneHancer: genome-wide integration of enhancers and target genes in GeneCards. Database J Biol Databases Curation. 2017;2017. doi: 10.1093/database/bax028 28605766 PMC5467550

[31] Khialeeva E, Carpenter EM. Nonneuronal roles for the reelin signaling pathway. Dev Dyn. 2017;246: 217–226. doi: 10.1002/dvdy.24462 27739126

[32] Racetin A, Jurić M, Filipović N, Šolić I, Kosović I, Durdov MG, et al. Expression and localization of DAB1 and Reelin during normal human kidney development. Croat Med J. 2019;60: 521–531. doi: 10.3325/cmj.2019.60.521 31894918 PMC6952895

[33] Ogino H, Hisanaga A, Kohno T, Kondo Y, Okumura K, Kamei T, et al. Secreted Metalloproteinase ADAMTS-3 Inactivates Reelin. J Neurosci Off J Soc Neurosci. 2017;37: 3181–3191. doi: 10.1523/JNEUROSCI.3632-16.2017 28213441 PMC6596773

[34] Freedman AH, Schweizer RM, Vecchyo DO-D, Han E, Davis BW, Gronau I, et al. Demographically-Based Evaluation of Genomic Regions under Selection in Domestic Dogs. PLOS Genet. 2016;12: e1005851. doi: 10.1371/journal.pgen.1005851 26943675 PMC4778760

[35] Wang G, Zhai W, Yang H, Fan R, Cao X, Zhong L, et al. The genomics of selection in dogs and the parallel evolution between dogs and humans. Nat Commun. 2013;4: 1860. doi: 10.1038/ncomms2814 23673645

[36] Balbas MD, Burgess MR, Murali R, Wongvipat J, Skaggs BJ, Mundel P, et al. MAGI-2 scaffold protein is critical for kidney barrier function. Proc Natl Acad Sci. 2014;111: 14876–14881. doi: 10.1073/pnas.1417297111 25271328 PMC4205655

[37] Shirata N, Ihara K-I, Yamamoto-Nonaka K, Seki T, Makino S-I, Oliva Trejo JA, et al. Glomerulosclerosis Induced by Deficiency of Membrane-Associated Guanylate Kinase Inverted 2 in Kidney Podocytes. J Am Soc Nephrol JASN. 2017;28: 2654–2669. doi: 10.1681/ASN.2016121356 28539383 PMC5576941

[38] Zuo Z, Shen J-X, Pan Y, Pu J, Li Y-G, Shao X-H, et al. Weighted Gene Correlation Network Analysis (WGCNA) Detected Loss of MAGI2 Promotes Chronic Kidney Disease (CKD) by Podocyte Damage. Cell Physiol Biochem Int J Exp Cell Physiol Biochem Pharmacol. 2018;51: 244–261. doi: 10.1159/000495205 30448842

[39] Kim H-G, Ahn J-W, Kurth I, Ullmann R, Kim H-T, Kulharya A, et al. WDR11, a WD Protein that Interacts with Transcription Factor EMX1, Is Mutated in Idiopathic Hypogonadotropic Hypogonadism and Kallmann Syndrome. Am J Hum Genet. 2010;87: 465–479. doi: 10.1016/j.ajhg.2010.08.018 20887964 PMC2948809

[40] Zenteno JC, Méndez JP, Maya-Núñez G, Ulloa-Aguirre A, Kofman-Alfaro S. Renal abnormalities in patients with Kallmann syndrome. BJU Int. 1999;83: 383–386. doi: 10.1046/j.1464-410x.1999.00027.x 10210557

[41] Sims-Lucas S, Cusack B, Baust J, Eswarakumar VP, Masatoshi H, Takeuchi A, et al. Fgfr1 and the IIIc isoform of Fgfr2 play critical roles in the metanephric mesenchyme mediating early inductive events in kidney development. Dev Dyn Off Publ Am Assoc Anat. 2011;240: 240–249. doi: 10.1002/dvdy.22501 21128305 PMC3093196

[42] Li X, Wang J, Li W, Xu Y, Shao D, Xie Y, et al. Characterization of ppGalNAc-T18, a member of the vertebrate-specific Y subfamily of UDP-N-acetyl-α-D-galactosamine:polypeptide N-acetylgalactosaminyltransferases. Glycobiology. 2012;22: 602–615. doi: 10.1093/glycob/cwr179 22171061

[43] Coit P, Ortiz-Fernandez L, Lewis EE, McCune WJ, Maksimowicz-McKinnon K, Sawalha AH. A longitudinal and transancestral analysis of DNA methylation patterns and disease activity in lupus patients. JCI Insight. 5: e143654. doi: 10.1172/jci.insight.143654 33108347 PMC7710270

[44] Fu Z-J, Wang Z-Y, Xu L, Chen X-H, Li X-X, Liao W-T, et al. HIF-1α-BNIP3-mediated mitophagy in tubular cells protects against renal ischemia/reperfusion injury. Redox Biol. 2020;36: 101671. doi: 10.1016/j.redox.2020.101671 32829253 PMC7452120

[45] Moyers BT, Morrell PL, McKay JK. Genetic Costs of Domestication and Improvement. J Hered. 2018;109: 103–116. doi: 10.1093/jhered/esx069 28992310

[46] Lindblad-Toh K, Wade CM, Mikkelsen TS, Karlsson EK, Jaffe DB, Kamal M, et al. Genome sequence, comparative analysis and haplotype structure of the domestic dog. Nature. 2005;438: 803–819. doi: 10.1038/nature04338 16341006

[47] Xu X, Weinstein M, Li C, Naski M, Cohen RI, Ornitz DM, et al. Fibroblast growth factor receptor 2 (FGFR2)-mediated reciprocal regulation loop between FGF8 and FGF10 is essential for limb induction. Dev Camb Engl. 1998;125: 753–765. doi: 10.1242/dev.125.4.753 9435295

[48] Miki T, Bottaro DP, Fleming TP, Smith CL, Burgess WH, Chan AM, et al. Determination of ligand-binding specificity by alternative splicing: two distinct growth factor receptors encoded by a single gene. Proc Natl Acad Sci U S A. 1992;89: 246–250. doi: 10.1073/pnas.89.1.246 1309608 PMC48213

[49] Revest JM, Spencer-Dene B, Kerr K, De Moerlooze L, Rosewell I, Dickson C. Fibroblast growth factor receptor 2-IIIb acts upstream of Shh and Fgf4 and is required for limb bud maintenance but not for the induction of Fgf8, Fgf10, Msx1, or Bmp4. Dev Biol. 2001;231: 47–62. doi: 10.1006/dbio.2000.0144 11180951

[50] Chambers BE, Gerlach GF, Clark EG, Chen KH, Levesque AE, Leshchiner I, et al. Tfap2a is a novel gatekeeper of nephron differentiation during kidney development. Development. 2019;146: dev172387. doi: 10.1242/dev.172387 31160420 PMC6633607

[51] Guan Y, Liu H, Ma Z, Li S-Y, Park J, Sheng X, et al. Dnmt3a and Dnmt3b-Decommissioned Fetal Enhancers are Linked to Kidney Disease. J Am Soc Nephrol. 2020;31: 765–782. doi: 10.1681/ASN.2019080797 32127410 PMC7191927

[52] Yoshida T, Kato K, Yokoi K, Oguri M, Watanabe S, Metoki N, et al. Association of gene polymorphisms with chronic kidney disease in Japanese individuals. Int J Mol Med. 2009;24: 539–547. doi: 10.3892/ijmm_00000263 19724895

[53] Xing J, He Y-C, Wang K-Y, Wan P-Z, Zhai X-Y. Involvement of YTHDF1 in renal fibrosis progression via up-regulating YAP. FASEB J. 2022;36: e22144. doi: 10.1096/fj.202100172RR 34990050

[54] Centini R, Tsang M, Iwata T, Park H, Delrow J, Margineantu D, et al. Loss of Fnip1 alters kidney developmental transcriptional program and synergizes with TSC1 loss to promote mTORC1 activation and renal cyst formation. PloS One. 2018;13: e0197973. doi: 10.1371/journal.pone.0197973 29897930 PMC5999084

[55] O’Neill DG, Elliott J, Church DB, McGreevy PD, Thomson PC, Brodbelt DC. Chronic kidney disease in dogs in UK veterinary practices: prevalence, risk factors, and survival. J Vet Intern Med. 2013;27: 814–821. doi: 10.1111/jvim.12090 23647231

[56] Coyne M, Szlosek D, Clements C, McCrann D, Olavessen L. Association between breed and renal biomarkers of glomerular filtration rate in dogs. Vet Rec. 2020;187: e82. doi: 10.1136/vr.105733 32611706 PMC7799420

[57] Yu Z, Jin J, Tin A, Köttgen A, Yu B, Chen J, et al. Polygenic Risk Scores for Kidney Function and Their Associations with Circulating Proteome, and Incident Kidney Diseases. J Am Soc Nephrol JASN. 2021; ASN.2020111599. doi: 10.1681/ASN.2020111599 34548389 PMC8638405

[58] Piras D, Lepori N, Cabiddu G, Pani A. How Genetics Can Improve Clinical Practice in Chronic Kidney Disease: From Bench to Bedside. J Pers Med. 2022;12: 193. doi: 10.3390/jpm12020193 35207681 PMC8875178

[59] Chang CC, Chow CC, Tellier LC, Vattikuti S, Purcell SM, Lee JJ. Second-generation PLINK: rising to the challenge of larger and richer datasets. GigaScience. 2015;4: 7. doi: 10.1186/s13742-015-0047-8 25722852 PMC4342193

[60] Manichaikul A, Mychaleckyj JC, Rich SS, Daly K, Sale M, Chen W-M. Robust relationship inference in genome-wide association studies. Bioinforma Oxf Engl. 2010;26: 2867–2873. doi: 10.1093/bioinformatics/btq559 20926424 PMC3025716

[61] Wang C, Wallerman O, Arendt M-L, Sundström E, Karlsson Å, Nordin J, et al. A novel canine reference genome resolves genomic architecture and uncovers transcript complexity. Commun Biol. 2021;4: 185. doi: 10.1038/s42003-021-01698-x 33568770 PMC7875987

[62] Baker LA, Momen M, McNally R, Berres ME, Binversie EE, Sample SJ, et al. Biologically Enhanced Genome-Wide Association Study Provides Further Evidence for Candidate Loci and Discovers Novel Loci That Influence Risk of Anterior Cruciate Ligament Rupture in a Dog Model. Front Genet. 2021;12: 593515. doi: 10.3389/fgene.2021.593515 33763109 PMC7982834

[63] Barrett JC, Fry B, Maller J, Daly MJ. Haploview: analysis and visualization of LD and haplotype maps. Bioinforma Oxf Engl. 2005;21: 263–265. doi: 10.1093/bioinformatics/bth457 15297300

[64] Md Vasimuddin, Misra S, Li H, Aluru S. Efficient Architecture-Aware Acceleration of BWA-MEM for Multicore Systems. 2019 IEEE International Parallel and Distributed Processing Symposium (IPDPS). 2019. pp. 314–324. doi: 10.1109/IPDPS.2019.00041

[65] Li H, Handsaker B, Wysoker A, Fennell T, Ruan J, Homer N, et al. The Sequence Alignment/Map format and SAMtools. Bioinforma Oxf Engl. 2009;25: 2078–2079. doi: 10.1093/bioinformatics/btp352 19505943 PMC2723002

[66] DePristo MA, Banks E, Poplin R, Garimella KV, Maguire JR, Hartl C, et al. A framework for variation discovery and genotyping using next-generation DNA sequencing data. Nat Genet. 2011;43: 491–498. doi: 10.1038/ng.806 21478889 PMC3083463

[67] Delaneau O, Marchini J, 1000 Genomes Project Consortium, 1000 Genomes Project Consortium. Integrating sequence and array data to create an improved 1000 Genomes Project haplotype reference panel. Nat Commun. 2014;5: 3934. doi: 10.1038/ncomms4934 25653097 PMC4338501

[68] Howie B, Fuchsberger C, Stephens M, Marchini J, Abecasis GR. Fast and accurate genotype imputation in genome-wide association studies through pre-phasing. Nat Genet. 2012;44: 955–959. doi: 10.1038/ng.2354 22820512 PMC3696580

[69] Ostrander EA, Wang G-D, Larson G, vonHoldt BM, Davis BW, Jagannathan V, et al. Dog10K: an international sequencing effort to advance studies of canine domestication, phenotypes and health. Natl Sci Rev. 2019;6: 810–824. doi: 10.1093/nsr/nwz049 31598383 PMC6776107

[70] Amin SB, Anderson KJ, Boudreau CE, Martinez-Ledesma E, Kocakavuk E, Johnson KC, et al. Comparative Molecular Life History of Spontaneous Canine and Human Gliomas. Cancer Cell. 2020;37: 243–257.e7. doi: 10.1016/j.ccell.2020.01.004 32049048 PMC7132629

[71] Marchant TW, Johnson EJ, McTeir L, Johnson CI, Gow A, Liuti T, et al. Canine Brachycephaly Is Associated with a Retrotransposon-Mediated Missplicing of SMOC2. Curr Biol CB. 2017;27: 1573–1584.e6. doi: 10.1016/j.cub.2017.04.057 28552356 PMC5462623

[72] Wan X, Yang C, Yang Q, Xue H, Fan X, Tang NLS, et al. BOOST: A Fast Approach to Detecting Gene-Gene Interactions in Genome-wide Case-Control Studies. Am J Hum Genet. 2010;87: 325–340. doi: 10.1016/j.ajhg.2010.07.021 20817139 PMC2933337

[73] Zhou Y, Zhou B, Pache L, Chang M, Khodabakhshi AH, Tanaseichuk O, et al. Metascape provides a biologist-oriented resource for the analysis of systems-level datasets. Nat Commun. 2019;10: 1523. doi: 10.1038/s41467-019-09234-6 30944313 PMC6447622

[74] vonHoldt BM, Pollinger JP, Lohmueller KE, Han E, Parker HG, Quignon P, et al. Genome-wide SNP and haplotype analyses reveal a rich history underlying dog domestication. Nature. 2010;464: 898–902. doi: 10.1038/nature08837 20237475 PMC3494089

[75] Axelsson E, Ratnakumar A, Arendt M-L, Maqbool K, Webster MT, Perloski M, et al. The genomic signature of dog domestication reveals adaptation to a starch-rich diet. Nature. 2013;495: 360–364. doi: 10.1038/nature11837 23354050

[76] Cingolani P, Platts A, Wang LL, Coon M, Nguyen T, Wang L, et al. A program for annotating and predicting the effects of single nucleotide polymorphisms, SnpEff: SNPs in the genome of Drosophila melanogaster strain w1118; iso-2; iso-3. Fly (Austin). 2012;6: 80–92. doi: 10.4161/fly.19695 22728672 PMC3679285

[77] Vietri Rudan M, Barrington C, Henderson S, Ernst C, Odom DT, Tanay A, et al. Comparative Hi-C Reveals that CTCF Underlies Evolution of Chromosomal Domain Architecture. Cell Rep. 2015;10: 1297–1309. doi: 10.1016/j.celrep.2015.02.004 25732821 PMC4542312

[78] Consortium Zoonomia. A comparative genomics multitool for scientific discovery and conservation. Nature. 2020;587: 240–245. doi: 10.1038/s41586-020-2876-6 33177664 PMC7759459

[79] ENCODE Project Consortium. An integrated encyclopedia of DNA elements in the human genome. Nature. 2012;489: 57–74. doi: 10.1038/nature11247 22955616 PMC3439153

