# Supplementary material for: Bayesian mixed model analysis uncovered 21 risk loci for chronic kidney disease in boxer dogs
Source: PLoS Genet. 2023 Jan 24;19(1):e1010599. doi: 10.1371/journal.pgen.1010599 (PMC9897549; doi:10.1371/journal.pgen.1010599)
Supplement: S1 Table — (DOCX) [file pgen.1010599.s001.docx]

S1 Table. Origin of boxers used in this study

| country | All | | After QC | |
| --- | --- | --- | --- | --- |
|  | case | control | case | control |
| Australia | 4 | 0 | 3 | 0 |
| Denmark | 5 | 2 | 1 | 1 |
| Finland | 21 | 21 | 12 | 17 |
| Germany | 1 | 0 | 1 | 0 |
| Norway | 64 | 101 | 45 | 68 |
| Sweden | 30 | 36 | 22 | 30 |
| UK | 24 | 19 | 21 | 15 |
| US | 19 | 14 | 12 | 6 |
