# Supplementary material for: Bayesian mixed model analysis uncovered 21 risk loci for chronic kidney disease in boxer dogs
Source: PLoS Genet. 2023 Jan 24;19(1):e1010599. doi: 10.1371/journal.pgen.1010599 (PMC9897549; doi:10.1371/journal.pgen.1010599)
Supplement: S2 Table — (DOCX) [file pgen.1010599.s002.docx]

S2 Table. Top 50 markers from Bayesian analysis

| chromosome | position | marker ID | reference_allele | alternative_allele | absolute effect size |
| --- | --- | --- | --- | --- | --- |
| chr18 | 16900760 | BICF2S23128680 | T | G | 2.96E-03 |
| chr18 | 15551821 | BICF2S23450214 | C | T | 2.17E-03 |
| chr28 | 31238605 | BICF2P865971 | G | A | 1.96E-03 |
| chr28 | 31266731 | BICF2P455596 | G | T | 1.82E-03 |
| chr28 | 31246911 | TIGRP2P360082 | A | G | 1.72E-03 |
| chr18 | 15583568 | TIGRP2P240182 | G | A | 1.62E-03 |
| chr18 | 15507722 | BICF2P963882 | T | C | 1.60E-03 |
| chr18 | 15597563 | BICF2S2336067 | T | C | 1.58E-03 |
| chr21 | 34800630 | BICF2S23054624 | C | T | 1.56E-03 |
| chr11 | 20249022 | BICF2G630296198 | C | G | 1.48E-03 |
| chr18 | 15320389 | BICF2P292067 | T | C | 1.46E-03 |
| chr18 | 15514249 | BICF2S23454133 | A | G | 1.46E-03 |
| chr21 | 34800399 | BICF2S23054623 | G | A | 1.16E-03 |
| chr21 | 35204790 | BICF2P412190 | C | T | 1.09E-03 |
| chr21 | 35192025 | BICF2P1188210 | G | A | 1.04E-03 |
| chr18 | 14348044 | BICF2P718938 | G | A | 9.93E-04 |
| chr5 | 65936980 | BICF2P515777 | T | C | 9.72E-04 |
| chr5 | 65922432 | BICF2P94251 | T | C | 9.37E-04 |
| chr5 | 65952204 | BICF2P56458 | T | A | 9.27E-04 |
| chr35 | 15219208 | BICF2P1261670 | C | T | 8.52E-04 |
| chr35 | 15216497 | TIGRP2P407227 | G | A | 8.13E-04 |
| chr11 | 17662230 | BICF2P686280 | C | T | 7.56E-04 |
| chr18 | 18020389 | BICF2P155823 | A | T | 7.52E-04 |
| chr20 | 16198625 | BICF2S23036843 | A | G | 7.48E-04 |
| chr2 | 36171796 | BICF2P720430 | G | A | 7.04E-04 |
| chr35 | 14974125 | TIGRP2P407148 | T | C | 6.81E-04 |
| chr35 | 14990212 | BICF2P506957 | A | G | 6.58E-04 |
| chr18 | 14437466 | BICF2S2435042 | A | G | 6.37E-04 |
| chr18 | 14269995 | BICF2S23421089 | T | C | 5.97E-04 |
| chr18 | 15398046 | TIGRP2P240132 | T | C | 5.87E-04 |
| chr18 | 14522674 | BICF2S23030029 | G | A | 5.84E-04 |
| chr20 | 16328816 | BICF2P483847 | C | T | 5.73E-04 |
| chr18 | 15153829 | BICF2P547535 | C | T | 5.61E-04 |
| chr13 | 62849434 | BICF2G630745991 | C | T | 5.59E-04 |
| chr18 | 18531233 | BICF2S22944761 | G | C | 5.43E-04 |
| chr18 | 14402572 | BICF2S22963174 | T | G | 5.23E-04 |
| chr13 | 62742887 | BICF2G630746191 | T | C | 5.20E-04 |
| chr24 | 47612182 | BICF2G630495908 | G | A | 5.18E-04 |
| chr28 | 40353980 | BICF2P820347 | C | T | 5.09E-04 |
| chr35 | 11220863 | BICF2G630773785 | T | C | 5.08E-04 |
| chr20 | 36969206 | BICF2P1410711 | T | C | 5.08E-04 |
| chr14 | 22230171 | BICF2P426684 | C | A | 5.06E-04 |
| chr36 | 9491520 | BICF2G630762815 | A | G | 5.02E-04 |
| chr30 | 14235980 | BICF2P1443519 | C | T | 4.92E-04 |
| chr14 | 50108428 | BICF2P969730 | A | G | 4.92E-04 |
| chr17 | 19445677 | BICF2P257298 | T | C | 4.78E-04 |
| chr18 | 17512924 | BICF2S23512155 | A | G | 4.76E-04 |
| chr36 | 9608263 | BICF2G630762689 | A | G | 4.73E-04 |
| chr3 | 2052050 | BICF2P1176410 | T | C | 4.68E-04 |
| chr18 | 17715346 | BICF2S2333289 | T | G | 4.67E-04 |
