# Supplementary material for: Bayesian mixed model analysis uncovered 21 risk loci for chronic kidney disease in boxer dogs
Source: PLoS Genet. 2023 Jan 24;19(1):e1010599. doi: 10.1371/journal.pgen.1010599 (PMC9897549; doi:10.1371/journal.pgen.1010599)
Supplement: S4 Table — (DOCX) [file pgen.1010599.s004.docx]

S4 Table. Summary of whole genome sequencing data of 20 Norwegian boxers

| Sample ID | Total paired-reads (M) | Aligned percentage | Coverage | phenotype | Accession |
| --- | --- | --- | --- | --- | --- |
| 10-Box-183 | 160.14 | 99.57% | 19.08 X | case | ERR6182498 |
| 11-Box-195 | 170.01 | 99.53% | 20.23 X | case | ERR6182485 |
| 14-BOX160 | 251.7 | 99.78% | 30.08 X | case | ERR6182491 |
| 15-BOX170 | 243.79 | 99.86% | 29.19 X | case | ERR6182494 |
| 16-BOX163 | 211.61 | 99.82% | 25.3 X | control | ERR6182492 |
| 17-BOX167 | 249.88 | 99.84% | 29.92 X | control | ERR6182493 |
| 1-Box-01 | 266.08 | 99.72% | 31.75 X | control | ERR6182485 |
| 20-BOX264 | 228.06 | 99.80% | 27 X | control | ERR6182500 |
| 21-BOX269 | 406.53 | 99.81% | 48.08 X | case | ERR6182501 |
| 26-BOX271 | 188.01 | 99.80% | 22.28 X | case | ERR6182502 |
| 27-BOX276 | 218.84 | 99.82% | 25.96 X | unknown | ERR6182503 |
| 2-Box-60 | 197.9 | 99.70% | 23.56 X | control | ERR6182486 |
| 3-Box-91 | 171.49 | 99.58% | 20.39 X | control | ERR6182487 |
| 4-Box-132 | 154.45 | 99.67% | 18.41 X | control | ERR6182488 |
| 5-Box-146 | 199.22 | 99.77% | 23.81 X | case | ERR6182489 |
| 6-Box-147 | 262.48 | 99.78% | 31.39 X | case | ERR6182490 |
| 7-Box-174 | 233.26 | 99.67% | 27.82 X | case | ERR6182495 |
| 8-Box-175 | 232.26 | 99.71% | 27.73 X | control | ERR6182496 |
| 9-Box-179 | 220.27 | 99.58% | 26.24 X | case | ERR6182497 |
| BOX282 | 254.87 | 99.89% | 29.88 X | case | ERR6182504 |
