# Supplementary material for: Bayesian mixed model analysis uncovered 21 risk loci for chronic kidney disease in boxer dogs
Source: PLoS Genet. 2023 Jan 24;19(1):e1010599. doi: 10.1371/journal.pgen.1010599 (PMC9897549; doi:10.1371/journal.pgen.1010599)
Supplement: S5 Table — (DOCX) [file pgen.1010599.s005.docx]

S5 Table. Average of risk allele load of 21 chronic kidney disease loci in 75 breeds

| Breed | Sample size | Mean risk load | Stdev. risk load |
| --- | --- | --- | --- |
| Bloodhound | 10 | 23 | 2 |
| Cavalier King Charles Spaniel | 10 | 22 | 1.9 |
| Collie | 10 | 22 | 2 |
| Flat Coated Retriever | 10 | 22 | 2.2 |
| Samoyed | 10 | 22 | 2.5 |
| Shar Pei | 10 | 22 | 1.7 |
| Australian Shepherd | 10 | 21 | 2.5 |
| Chow Chow | 10 | 21 | 2.4 |
| Leonberger | 10 | 21 | 2.5 |
| Akita | 10 | 20 | 2.2 |
| Australian Terrier | 10 | 20 | 2.3 |
| Border Collie | 10 | 20 | 2.4 |
| Bullmastiff | 10 | 20 | 2.7 |
| Foxhound | 10 | 20 | 3.7 |
| Havanese | 10 | 20 | 1.7 |
| Irish Water Spaniel | 10 | 20 | 2.6 |
| Shih Tzu | 10 | 20 | 1.9 |
| Siberian Husky | 10 | 20 | 1.8 |
| Standard Poodle | 10 | 20 | 2.9 |
| Basset Hound | 10 | 19 | 1.5 |
| Golden Retriever | 10 | 19 | 3.1 |
| Pembroke Welsh Corgi | 10 | 19 | 2.5 |
| Shetland Sheepdog | 10 | 19 | 2.6 |
| Rottweiler | 11 | 18 | 2.4 |
| Alaskan Malamute | 10 | 18 | 2.4 |
| Basenji | 10 | 18 | 1.4 |
| Beagle | 10 | 18 | 1.7 |
| Borzoi | 10 | 18 | 3.1 |
| Boston Terrier | 10 | 18 | 2.1 |
| Cardigan Welsh Corgi | 10 | 18 | 1.4 |
| Chihuahua | 10 | 18 | 2.1 |
| Doberman Pinscher | 10 | 18 | 2.2 |
| French Bulldog | 10 | 18 | 2.2 |
| Giant Schnauzer | 10 | 18 | 2.4 |
| Greyhound | 10 | 18 | 2.1 |
| Miniature Bull Terrier | 10 | 18 | 1.7 |
| Miniature Pinscher | 10 | 18 | 1.7 |
| Miniature Schnauzer | 10 | 18 | 2.1 |
| Norwich Terrier | 10 | 18 | 1.4 |
| Pekingese | 10 | 18 | 2.5 |
| Petit Basset Griffon Vendeen | 10 | 18 | 2.5 |
| Pug | 10 | 18 | 1.5 |
| Whippet | 10 | 18 | 2.2 |
| Mastiff | 12 | 17 | 1.8 |
| Afghan Hound | 10 | 17 | 1.2 |
| American Hairless Terrier | 10 | 17 | 2.7 |
| Boxer | 10 | 17 | 2.2 |
| Brittany | 10 | 17 | 3.4 |
| Bull Terrier | 10 | 17 | 1.7 |
| Dachshund | 10 | 17 | 2.7 |
| English Springer Spaniel | 10 | 17 | 2.1 |
| Kuvasz | 10 | 17 | 1.3 |
| Labrador Retriever | 10 | 17 | 2.6 |
| Scottish Deerhound | 10 | 17 | 2.3 |
| Toy Poodle | 10 | 17 | 2.1 |
| Yorkshire Terrier | 10 | 17 | 2 |
| Belgian Tervuren | 10 | 16 | 1.6 |
| Great Dane | 10 | 16 | 2.5 |
| Keeshond | 10 | 16 | 2.1 |
| Miniature Poodle | 10 | 16 | 2.6 |
| Papillon | 10 | 16 | 2.9 |
| Saluki | 10 | 16 | 3.1 |
| Belgian Sheepdog | 10 | 15 | 1.6 |
| Briard | 10 | 15 | 1.3 |
| Cairn Terrier | 10 | 15 | 2.4 |
| German Shorthaired Pointer | 10 | 15 | 2.3 |
| Newfoundland | 10 | 15 | 1.9 |
| Old English Sheepdog | 10 | 15 | 3.5 |
| Pomeranian | 10 | 15 | 1.8 |
| Staffordshire Bull Terrier | 10 | 15 | 2.6 |
| American Cocker Spaniel | 10 | 14 | 1.9 |
| Irish Wolfhound | 10 | 14 | 1.8 |
| Scottish Terrier | 10 | 14 | 2.1 |
| West Highland White Terrier | 10 | 14 | 2.5 |
| German Shepherd Dog | 10 | 12 | 2.5 |
