# Supplementary material for: Bayesian mixed model analysis uncovered 21 risk loci for chronic kidney disease in boxer dogs
Source: PLoS Genet. 2023 Jan 24;19(1):e1010599. doi: 10.1371/journal.pgen.1010599 (PMC9897549; doi:10.1371/journal.pgen.1010599)
Supplement: S6 Table — (DOCX) [file pgen.1010599.s006.docx]

S6 Table. ANOVA test of 21 BayesR markers with the chronic kidney disease

| Marker | Df | Sum Square | Mean Square | F value | Pr(>F) |
| --- | --- | --- | --- | --- | --- |
| BICF2G630296198 | 2 | 2.602 | 1.3011 | 10.116 | 6.77E-05 |
| BICF2G630495908 | 2 | 1.57 | 0.7848 | 6.102 | 0.002715 |
| BICF2G630745991 | 2 | 4.681 | 2.3406 | 18.199 | 6.09E-08 |
| BICF2G630762815 | 2 | 2.83 | 1.415 | 11.003 | 3.06E-05 |
| BICF2G630773785 | 2 | 3.384 | 1.692 | 13.156 | 4.55E-06 |
| BICF2P1176410 | 1 | 1.017 | 1.0171 | 7.908 | 0.005452 |
| BICF2P1261670 | 2 | 2.215 | 1.1075 | 8.611 | 0.000266 |
| BICF2P1410711 | 1 | 0.126 | 0.1255 | 0.976 | 0.324504 |
| BICF2P1443519 | 2 | 0.794 | 0.3968 | 3.085 | 0.048079 |
| BICF2P257298 | 2 | 1.969 | 0.9843 | 7.653 | 0.000641 |
| BICF2P426684 | 2 | 0.718 | 0.3588 | 2.79 | 0.064038 |
| BICF2P515777 | 1 | 1.182 | 1.1818 | 9.189 | 0.002783 |
| BICF2P686280 | 2 | 0.617 | 0.3084 | 2.398 | 0.093746 |
| BICF2P720430 | 2 | 1.369 | 0.6843 | 5.32 | 0.005667 |
| BICF2P820347 | 2 | 0.507 | 0.2534 | 1.97 | 0.142327 |
| BICF2P865971 | 2 | 0.368 | 0.184 | 1.431 | 0.241736 |
| BICF2P969730 | 2 | 1.067 | 0.5333 | 4.146 | 0.017316 |
| BICF2S23036843 | 2 | 2.793 | 1.3966 | 10.859 | 3.47E-05 |
| BICF2S23054624 | 2 | 0.702 | 0.3511 | 2.73 | 0.067866 |
| BICF2S23128680 | 2 | 1.281 | 0.6406 | 4.981 | 0.007817 |
| TIGRP2P407148 | 2 | 0.178 | 0.0889 | 0.691 | 0.502207 |
| Residuals | 185 | 23.793 | 0.1286 |  |  |
