# Supplementary material for: Bayesian mixed model analysis uncovered 21 risk loci for chronic kidney disease in boxer dogs
Source: PLoS Genet. 2023 Jan 24;19(1):e1010599. doi: 10.1371/journal.pgen.1010599 (PMC9897549; doi:10.1371/journal.pgen.1010599)
Supplement: S7 Table — (DOCX) [file pgen.1010599.s007.docx]

S7 Table. Selection signals from domestication found around the chronic kidney disease regions

| CKD region | | | selective region | | | Distance(bp) | Source |
| --- | --- | --- | --- | --- | --- | --- | --- |
| chromosome | start | end | chromosome | start | end |  |  |
| chr18 | 13894632 | 19137370 | chr18 | 15776035 | 16066130 | 0 | Freedman, 2016[1] Wang, 2013[2] |
| chr28 | 30794656 | 31398277 | chr28 | 31111410 | 31242063 | 0 | Freedman, 2016[1] |
| chr2 | 36171795 | 36346340 | chr2 | 35817958 | 35937943 | 233853 | Wang, 2013[2] |
| chr14 | 49731336 | 50705080 | chr14 | 49353706 | 49475266 | 256071 | Freedman, 2016[1] |
| chr11 | 19343187 | 20249022 | chr11 | 18950326 | 19050344 | 292844 | Wang, 2013[2] |

References

1. Freedman AH, Schweizer RM, Vecchyo DO-D, Han E, Davis BW, Gronau I, et al. Demographically-Based Evaluation of Genomic Regions under Selection in Domestic Dogs. PLOS Genet. 2016;12: e1005851. doi:10.1371/journal.pgen.1005851

2. Wang G, Zhai W, Yang H, Fan R, Cao X, Zhong L, et al. The genomics of selection in dogs and the parallel evolution between dogs and humans. Nat Commun. 2013;4: 1860. doi:10.1038/ncomms2814
