# Supplementary material for: Bayesian mixed model analysis uncovered 21 risk loci for chronic kidney disease in boxer dogs
Source: PLoS Genet. 2023 Jan 24;19(1):e1010599. doi: 10.1371/journal.pgen.1010599 (PMC9897549; doi:10.1371/journal.pgen.1010599)
Supplement: S8 Table — (DOCX) [file pgen.1010599.s008.docx]

S8 Table. Location of 5,206 imputed variants in chronic kidney disease regions

| Location* | counts |
| --- | --- |
| 3 prime UTR variant | 24 |
| 5 prime UTR variant | 4 |
| downstream gene variant | 361 |
| intergenic region | 2443 |
| intragenic variant | 29 |
| intron variant | 2692 |
| missense variant | 4 |
| non coding transcript exon variant | 34 |
| splice region variant&intron variant | 5 |
| splice region variant&non coding transcript exon variant | 1 |
| synonymous variant | 11 |
| upstream gene variant | 342 |

* Locations were annotated using SNPEFF based on the transcripts from NCBI annotation of CanFam4. One variant could be annotated with multiple location types, depending on the transcripts.
