# Supplementary material for: Bayesian mixed model analysis uncovered 21 risk loci for chronic kidney disease in boxer dogs
Source: PLoS Genet. 2023 Jan 24;19(1):e1010599. doi: 10.1371/journal.pgen.1010599 (PMC9897549; doi:10.1371/journal.pgen.1010599)
Supplement: S9 Table — (DOCX) [file pgen.1010599.s009.docx]

S9 Table. Missense mutations detected in chronic kidney disease regions

| chromosome | position | reference allele | alternative allele | affected Gene | Transcript id | strand | Nucleotide change | amino acid change | PROVEAN score | PROVEAN prediction |
| --- | --- | --- | --- | --- | --- | --- | --- | --- | --- | --- |
| chr13 | 63105998 | A | C | ANKRD17 | XM_038556303.1 | minus | c.152T>G | p.Val51Gly | 0.245 | Neutral |
|  |  |  |  |  | XM_038556304.1 | minus | c.152T>G | p.Val51Gly | 0.245 | Neutral |
|  |  |  |  |  | XM_038556308.1 | minus | c.152T>G | p.Val51Gly | 0.17 | Neutral |
|  |  |  |  |  | XM_038556309.1 | minus | c.152T>G | p.Val51Gly | 0.17 | Neutral |
|  |  |  |  |  | XM_038556307.1 | minus | c.152T>G | p.Val51Gly | 0.17 | Neutral |
| chr17 | 18761305 | C | G | ITSN2 | XM_038560916.1 | minus | c.1784G>C | p.Arg595Thr | -3.116 | Deleterious |
| chr18 | 15529106 | T | C | KMT2E | XM_038562741.1 | minus | c.4087A>G | p.Met1363Val | 0.183 | Neutral |
|  |  |  |  |  | XM_038562744.1 | minus | c.3649A>G | p.Met1217Val | 0.27 | Neutral |
|  |  |  |  |  | XM_038562742.1 | minus | c.4087A>G | p.Met1363Val | 0.183 | Neutral |
|  |  |  |  |  | XM_038562743.1 | minus | c.3961A>G | p.Met1321Val | 0.103 | Neutral |
| chr18 | 18267721 | A | G | MAGI2 | XM_038562824.1 | minus | c.3931T>C | p.Ser1311Pro | -0.958 | Neutral |
|  |  |  |  |  | XM_038562825.1 | minus | c.3889T>C | p.Ser1297Pro | -0.955 | Neutral |
