# Supplementary material for: Bayesian mixed model analysis uncovered 21 risk loci for chronic kidney disease in boxer dogs
Source: PLoS Genet. 2023 Jan 24;19(1):e1010599. doi: 10.1371/journal.pgen.1010599 (PMC9897549; doi:10.1371/journal.pgen.1010599)
Supplement: S10 Table — (DOCX) [file pgen.1010599.s010.docx]

S10 Table. Seventeen variants identified with putative regulatory function

| ID | chr | position | phyloP | hg38_chr | hg38_position | cCRE | GeneHancer | cell lines with HS | Reference allele | Alternative allele | Risk allele |
| --- | --- | --- | --- | --- | --- | --- | --- | --- | --- | --- | --- |
| C1 | chr14 | 50263991 | 3.00 | chr7 | 110623888 | EH38E2581379 | - | - | C | T | C |
| C2 | chr17 | 19415442 | 2.97 | chr2 | 25062537 | EH38E1980950 | - | 6 | A | G | A |
| C3 | chr18 | 15356679 | 1.00 | chr7 | 105306568 | EH38E2578548 | GH07J105306 | 35 | C | G | C |
| C4 | chr18 | 16949830 | 2.92 | chr7 | 103569880 | EH38E2577730 | - | 8 | G | C | G |
| C5 | chr18 | 17885672 | 4.53 | chr7 | 77536644 | EH38E2565273 | GH07J077535 | 4 | G | A | G |
| C6 | chr18 | 18116871 | 5.87 | chr7 | 77840085 | EH38E2565490 | - | 4 | G | A | G |
| C7 | chr18 | 18257320 | 3.20 | chr7 | 78001395 | EH38E2565581 | - | - | A | G | A |
| C8 | chr18 | 18518972 | 1.48 | chr7 | 78301516 | EH38E2565734 | - | 41 | T | C | T |
| C9 | chr18 | 18551348 | 6.24 | chr7 | 78346053 | EH38E2565751 | - | - | A | G | A |
| C10 | chr18 | 18602288 | 3.45 | chr1 | 112517383 | EH38E1377031 | - | 8 | A | G | A |
| C11 | chr20 | 16290847 | 5.60 | chr3 | 1092898 | EH38E2173774 | GH03J001092 | 23 | G | A | A |
| C12 | chr21 | 34776034 | 2.65 | chr11 | 11056574 | EH38E1520515 | - | 2 | G | A | G |
| C13 | chr21 | 35197104 | 5.02 | chr11 | 11502587 | EH38E1520796 | - | 17 | C | T | C |
| C14 | chr28 | 40201268 | 4.25 | chr10 | 131426485 | EH38E1511661 | - | - | A | G | A |
| C15 | chr30 | 14288732 | 6.25 | chr15 | 47718881 | - | - | 72 | A | G | A |
| C16 | chr35 | 15301992 | 8.90 | chr6 | 14690749 | EH38E2448468 | - | 13 | C | T | C |
| C17 | chr36 | 9386570 | 1.00 | chr2 | 163778632 | EH38E2047281 | - | 69 | A | C | A |
