# Supplementary material for: Bayesian mixed model analysis uncovered 21 risk loci for chronic kidney disease in boxer dogs
Source: PLoS Genet. 2023 Jan 24;19(1):e1010599. doi: 10.1371/journal.pgen.1010599 (PMC9897549; doi:10.1371/journal.pgen.1010599)
Supplement: S11 Table — (DOCX) [file pgen.1010599.s011.docx]

S11 Table. Diagnosis of chronic kidney disease in boxers

| Diagnosis | Total | Case | Control |
| --- | --- | --- | --- |
| i) Clinical data | 123 | 52 | 71 |
| ii) Clinical data + Clinical chemistry | 78 | 33 | 45 |
| iii) Morphology | 53 | 32 | 21 |
| Total | 254 | 117 | 137 |
