# Supplementary material for: Bayesian mixed model analysis uncovered 21 risk loci for chronic kidney disease in boxer dogs
Source: PLoS Genet. 2023 Jan 24;19(1):e1010599. doi: 10.1371/journal.pgen.1010599 (PMC9897549; doi:10.1371/journal.pgen.1010599)
Supplement: S13 Table — (DOCX) [file pgen.1010599.s013.docx]

S13 Table. Cross validation of imputation with seven boxers.

| sample | sequencing depth | Called WGS genotypes | Missing imputed genotype | mistake type | | | mistake count | imputation rate | imputation accuracy |
| --- | --- | --- | --- | --- | --- | --- | --- | --- | --- |
|  |  |  |  | AA->AB | AA->BB | AB->AA |  |  |  |
| ERR2196023 | 28.26 X | 6848009 | 311973 | 66189 | 3486 | 127329 | 197004 | 96% | 97% |
| SRR10351592 | 26.56 X | 6352136 | 378889 | 105437 | 11953 | 263551 | 380941 | 95% | 95% |
| SRR15734886 | 17.83 X | 6460880 | 254646 | 47376 | 3786 | 114905 | 166067 | 97% | 98% |
| SRR8541911 | 22.72 X | 6163896 | 340347 | 104680 | 10485 | 166871 | 282036 | 95% | 96% |
| SRR8541912 | 20.23 X | 6135829 | 272792 | 71553 | 9932 | 126371 | 207856 | 96% | 97% |
| SRR8541917 | 22.71 X | 6101216 | 245121 | 60204 | 13497 | 112332 | 186033 | 97% | 98% |
| SRR8541918 | 22.5 X | 6146301 | 326388 | 104513 | 23070 | 168524 | 296107 | 96% | 96% |
