# Supplementary material for: Bayesian mixed model analysis uncovered 21 risk loci for chronic kidney disease in boxer dogs
Source: PLoS Genet. 2023 Jan 24;19(1):e1010599. doi: 10.1371/journal.pgen.1010599 (PMC9897549; doi:10.1371/journal.pgen.1010599)
Supplement: S14 Table — (DOCX) [file pgen.1010599.s014.docx]

S14 Table. Primer sequences for the EMSA assays

| ID* | Chr | Position | primer_id | primer_sequence |
| --- | --- | --- | --- | --- |
| C1 | chr14 | 50263991 | A1_risk_bio | /5Biosg/CTGACAAAATTCTTGCTTGATGCACAGTGTA |
|  |  |  | A1_risk_F | CTGACAAAATTCTTGCTTGATGCACAGTGTA |
|  |  |  | A1_risk_R | TACACTGTGCATCAAGCAAGAATTTTGTCAG |
|  |  |  | A1_prot_bio | /5Biosg/CTGACAAAATTCTTGTTTGATGCACAGTGTA |
|  |  |  | A1_prot_F | CTGACAAAATTCTTGTTTGATGCACAGTGTA |
|  |  |  | A1_prot_R | TACACTGTGCATCAAACAAGAATTTTGTCAG |
| C2 | chr17 | 19415442 | B1_risk_bio | /5Biosg/GTCGGTGTGCCAGGAAACAGCCAAGGACCTG |
|  |  |  | B1_risk_F | GTCGGTGTGCCAGGAAACAGCCAAGGACCTG |
|  |  |  | B1_risk_R | CAGGTCCTTGGCTGTTTCCTGGCACACCGAC |
|  |  |  | B1_prot_bio | /5Biosg/GTCGGTGTGCCAGGAGACAGCCAAGGACCTG |
|  |  |  | B1_prot_F | GTCGGTGTGCCAGGAGACAGCCAAGGACCTG |
|  |  |  | B1_prot_R | CAGGTCCTTGGCTGTCTCCTGGCACACCGAC |
| C3 | chr18 | 15356679 | C1_risk_bio | /5Biosg/TCTTTGTTTAAGACAGAGTAACATCCTGCTG |
|  |  |  | C1_risk_F | TCTTTGTTTAAGACAGAGTAACATCCTGCTG |
|  |  |  | C1_risk_R | CAGCAGGATGTTACTCTGTCTTAAACAAAGA |
|  |  |  | C1_prot_bio | /5Biosg/TCTTTGTTTAAGACACAGTAACATCCTGCTG |
|  |  |  | C1_prot_F | TCTTTGTTTAAGACACAGTAACATCCTGCTG |
|  |  |  | C1_prot_R | CAGCAGGATGTTACTGTGTCTTAAACAAAGA |
| C4 | chr18 | 16949830 | C2_risk_bio | /5Biosg/TGTAATTTAGAGAGAGAAATGTACATGCCCA |
|  |  |  | C2_risk_F | TGTAATTTAGAGAGAGAAATGTACATGCCCA |
|  |  |  | C2_risk_R | TGGGCATGTACATTTCTCTCTCTAAATTACA |
|  |  |  | C2_prot_bio | /5Biosg/TGTAATTTAGAGAGACAAATGTACATGCCCA |
|  |  |  | C2_prot_F | TGTAATTTAGAGAGACAAATGTACATGCCCA |
|  |  |  | C2_prot_R | TGGGCATGTACATTTGTCTCTCTAAATTACA |
| C5 | chr18 | 17885672 | C3_risk_bio | /5Biosg/GGGGGACGCGACTACGGTTCCCGGCATGCCT |
|  |  |  | C3_risk_F | GGGGGACGCGACTACGGTTCCCGGCATGCCT |
|  |  |  | C3_risk_R | AGGCATGCCGGGAACCGTAGTCGCGTCCCCC |
|  |  |  | C3_prot_bio | /5Biosg/GGGGGACGCGACTACAGTTCCCGGCATGCCT |
|  |  |  | C3_prot_F | GGGGGACGCGACTACAGTTCCCGGCATGCCT |
|  |  |  | C3_prot_R | AGGCATGCCGGGAACTGTAGTCGCGTCCCCC |
| C6 | chr18 | 18116871 | C4_risk_bio | /5Biosg/TGCTAAAATAGTAATAAGGAAGGGTGTAATA |
|  |  |  | C4_risk_F | TGCTAAAATAGTAATAAGGAAGGGTGTAATA |
|  |  |  | C4_risk_R | TATTACACCCTTCCTTATTACTATTTTAGCA |
|  |  |  | C4_prot_bio | /5Biosg/TGCTAAAATAGTAATGAGGAAGGGTGTAATA |
|  |  |  | C4_prot_F | TGCTAAAATAGTAATGAGGAAGGGTGTAATA |
|  |  |  | C4_prot_R | TATTACACCCTTCCTCATTACTATTTTAGCA |
| C7 | chr18 | 18257320 | C5_risk_bio | /5Biosg/AGGCACACAGCTCGTGCTAGCTAAATGCTAA |
|  |  |  | C5_risk_F | AGGCACACAGCTCGTGCTAGCTAAATGCTAA |
|  |  |  | C5_risk_R | TTAGCATTTAGCTAGCACGAGCTGTGTGCCT |
|  |  |  | C5_prot_bio | /5Biosg/AGGCACACAGCTCGTACTAGCTAAATGCTAA |
|  |  |  | C5_prot_F | AGGCACACAGCTCGTACTAGCTAAATGCTAA |
|  |  |  | C5_prot_R | TTAGCATTTAGCTAGTACGAGCTGTGTGCCT |
| C8 | chr18 | 18518972 | C6_risk_bio | /5Biosg/AATGCAACACCAGTCTGCTTATGCTTTCCCT |
|  |  |  | C6_risk_F | AATGCAACACCAGTCTGCTTATGCTTTCCCT |
|  |  |  | C6_risk_R | AGGGAAAGCATAAGCAGACTGGTGTTGCATT |
|  |  |  | C6_prot_bio | /5Biosg/AATGCAACACCAGTCCGCTTATGCTTTCCCT |
|  |  |  | C6_prot_F | AATGCAACACCAGTCCGCTTATGCTTTCCCT |
|  |  |  | C6_prot_R | AGGGAAAGCATAAGCGGACTGGTGTTGCATT |
| C9 | chr18 | 18551348 | C7_risk_bio | /5Biosg/ATGTGGCTGAAAAATAAAATTTCAAATTAGG |
|  |  |  | C7_risk_F | ATGTGGCTGAAAAATAAAATTTCAAATTAGG |
|  |  |  | C7_risk_R | CCTAATTTGAAATTTTATTTTTCAGCCACAT |
|  |  |  | C7_prot_bio | /5Biosg/ATGTGGCTGAAAAATGAAATTTCAAATTAGG |
|  |  |  | C7_prot_F | ATGTGGCTGAAAAATGAAATTTCAAATTAGG |
|  |  |  | C7_prot_R | CCTAATTTGAAATTTCATTTTTCAGCCACAT |
| C10 | chr18 | 18602288 | C8_risk_bio | /5Biosg/GTGCTTAGGGAATCTGCAGCCTTATCCAAGA |
|  |  |  | C8_risk_F | GTGCTTAGGGAATCTGCAGCCTTATCCAAGA |
|  |  |  | C8_risk_R | TCTTGGATAAGGCTGCAGATTCCCTAAGCAC |
|  |  |  | C8_prot_bio | /5Biosg/GTGCTTAGGGAATCTACAGCCTTATCCAAGA |
|  |  |  | C8_prot_F | GTGCTTAGGGAATCTACAGCCTTATCCAAGA |
|  |  |  | C8_prot_R | TCTTGGATAAGGCTGTAGATTCCCTAAGCAC |
| C11 | chr20 | 16290847 | D1_risk_bio | /5Biosg/CGCTCCCCTCCTGCTACATTCCTGACGGAAC |
|  |  |  | D1_risk_F | CGCTCCCCTCCTGCTACATTCCTGACGGAAC |
|  |  |  | D1_risk_R | GTTCCGTCAGGAATGTAGCAGGAGGGGAGCG |
|  |  |  | D1_prot_bio | /5Biosg/CGCTCCCCTCCTGCTGCATTCCTGACGGAAC |
|  |  |  | D1_prot_F | CGCTCCCCTCCTGCTGCATTCCTGACGGAAC |
|  |  |  | D1_prot_R | GTTCCGTCAGGAATGCAGCAGGAGGGGAGCG |
| C12 | chr21 | 34776034 | E1_risk_bio | /5Biosg/AGATTTAAAGAGTTAAAAGTCCATTAGGTAG |
|  |  |  | E1_risk_F | AGATTTAAAGAGTTAAAAGTCCATTAGGTAG |
|  |  |  | E1_risk_R | CTACCTAATGGACTTTTAACTCTTTAAATCT |
|  |  |  | E1_prot_bio | /5Biosg/AGATTTAAAGAGTTAGAAGTCCATTAGGTAG |
|  |  |  | E1_prot_F | AGATTTAAAGAGTTAGAAGTCCATTAGGTAG |
|  |  |  | E1_prot_R | CTACCTAATGGACTTCTAACTCTTTAAATCT |
| C13 | chr21 | 35197104 | E2_risk_bio | /5Biosg/GCCTGCTGCGTAATTTATCCATTGTGGTTCT |
|  |  |  | E2_risk_F | GCCTGCTGCGTAATTTATCCATTGTGGTTCT |
|  |  |  | E2_risk_R | AGAACCACAATGGATAAATTACGCAGCAGGC |
|  |  |  | E2_prot_bio | /5Biosg/GCCTGCTGCGTAATTCATCCATTGTGGTTCT |
|  |  |  | E2_prot_F | GCCTGCTGCGTAATTCATCCATTGTGGTTCT |
|  |  |  | E2_prot_R | AGAACCACAATGGATGAATTACGCAGCAGGC |
| C14 | chr28 | 40201268 | F1_risk_bio | /5Biosg/TTATAGCTGGTACCAACGGCTTCTGGCCCAA |
|  |  |  | F1_risk_F | TTATAGCTGGTACCAACGGCTTCTGGCCCAA |
|  |  |  | F1_risk_R | TTGGGCCAGAAGCCGTTGGTACCAGCTATAA |
|  |  |  | F1_prot_bio | /5Biosg/TTATAGCTGGTACCAGCGGCTTCTGGCCCAA |
|  |  |  | F1_prot_F | TTATAGCTGGTACCAGCGGCTTCTGGCCCAA |
|  |  |  | F1_prot_R | TTGGGCCAGAAGCCGCTGGTACCAGCTATAA |
| C15 | chr30 | 14288732 | G1_risk_bio | /5Biosg/AGCAGAAGGTAGGGGGACTTTCACTTCCCGT |
|  |  |  | G1_risk_F | AGCAGAAGGTAGGGGGACTTTCACTTCCCGT |
|  |  |  | G1_risk_R | ACGGGAAGTGAAAGTCCCCCTACCTTCTGCT |
|  |  |  | G1_prot_bio | /5Biosg/AGCAGAAGGTAGGGGAACTTTCACTTCCCGT |
|  |  |  | G1_prot_F | AGCAGAAGGTAGGGGAACTTTCACTTCCCGT |
|  |  |  | G1_prot_R | ACGGGAAGTGAAAGTTCCCCTACCTTCTGCT |
| C16 | chr35 | 15301992 | H1_risk_bio | /5Biosg/CTATTTTGCATTTTTTAGTAGAATTGTTTCA |
|  |  |  | H1_risk_F | CTATTTTGCATTTTTTAGTAGAATTGTTTCA |
|  |  |  | H1_risk_R | TGAAACAATTCTACTAAAAAATGCAAAATAG |
|  |  |  | H1_prot_bio | /5Biosg/CTATTTTGCATTTTTCAGTAGAATTGTTTCA |
|  |  |  | H1_prot_F | CTATTTTGCATTTTTCAGTAGAATTGTTTCA |
|  |  |  | H1_prot_R | TGAAACAATTCTACTGAAAAATGCAAAATAG |
| C17 | chr36 | 9386570 | I1_risk_bio | /5Biosg/AGTAAGGGCAAAGACAAATTTAAATCTGAGT |
|  |  |  | I1_risk_F | AGTAAGGGCAAAGACAAATTTAAATCTGAGT |
|  |  |  | I1_risk_R | ACTCAGATTTAAATTTGTCTTTGCCCTTACT |
|  |  |  | I1_prot_bio | /5Biosg/AGTAAGGGCAAAGACCAATTTAAATCTGAGT |
|  |  |  | I1_prot_F | AGTAAGGGCAAAGACCAATTTAAATCTGAGT |
|  |  |  | I1_prot_R | ACTCAGATTTAAATTGGTCTTTGCCCTTACT |

*Information of variants is available in S9 Table
