# Supplementary material for: Bayesian mixed model analysis uncovered 21 risk loci for chronic kidney disease in boxer dogs
Source: PLoS Genet. 2023 Jan 24;19(1):e1010599. doi: 10.1371/journal.pgen.1010599 (PMC9897549; doi:10.1371/journal.pgen.1010599)
Supplement: S1 Fig — The top 50 markers (blue dots; corresponding to a threshold of 0.000467, grey dash line) from Bayesian association analysis were from 15 different autosomes (chromosome numbers were labeled on the right side of each panel). (DOCX) [file pgen.1010599.s015.docx]

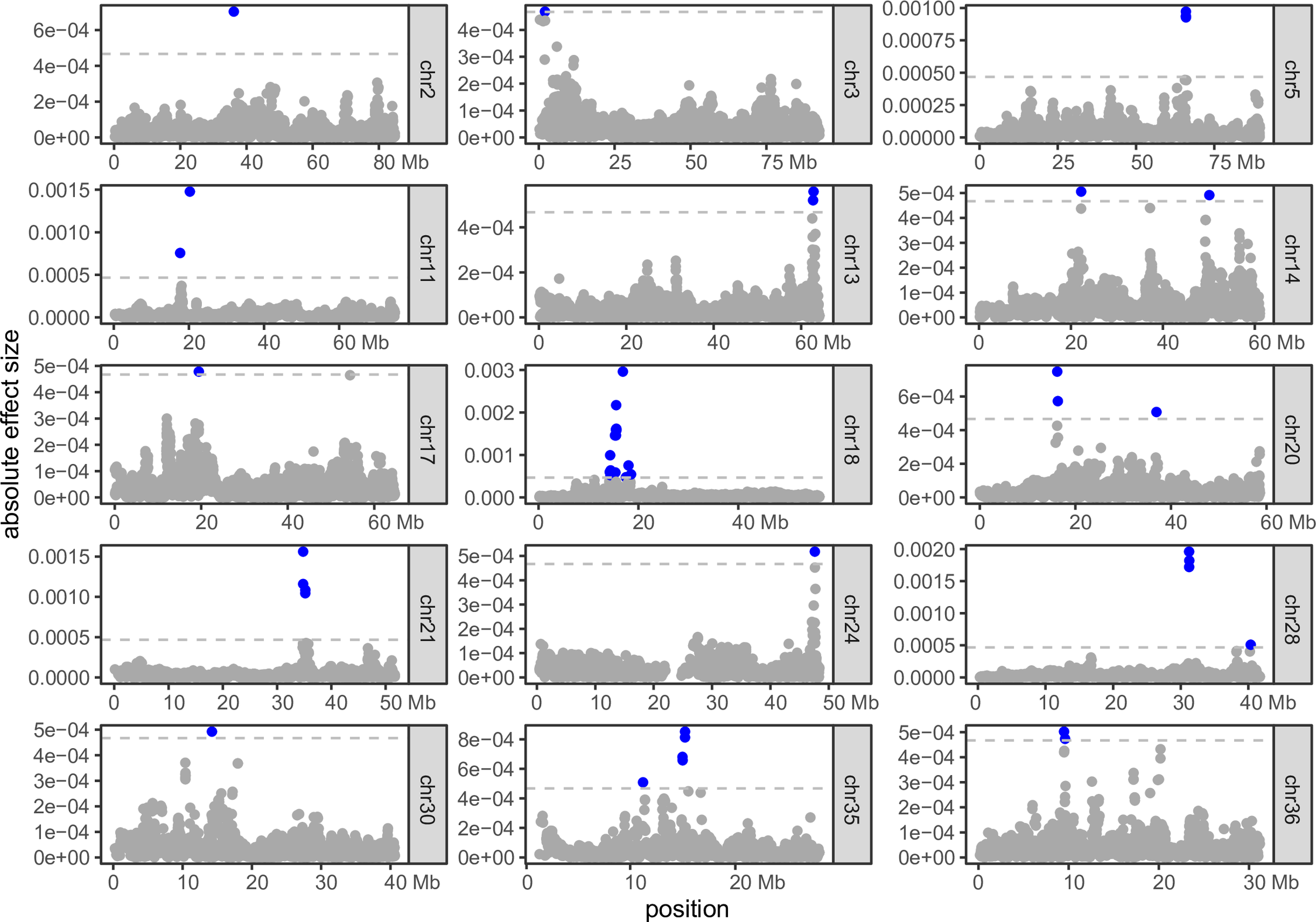


**S1 Fig. Absolute effect of top 50 BayesR markers from 15 autosomes.** The top 50 markers (blue dots; corresponding to a threshold of 0.000467, grey dash line) from Bayesian association analysis were from 15 different autosomes (chromosome numbers were labeled on the right side of each panel).
