# Supplementary material for: Bayesian mixed model analysis uncovered 21 risk loci for chronic kidney disease in boxer dogs
Source: PLoS Genet. 2023 Jan 24;19(1):e1010599. doi: 10.1371/journal.pgen.1010599 (PMC9897549; doi:10.1371/journal.pgen.1010599)
Supplement: S2 Fig — Imputed SNPs in LD with top markers from Bayesian analysis were compared with the candidate Cis-Regulatory Elements (cCRE, ENCODE), promotor and enhancer elements (GeneHancer), and Hypersensitivity (HS) signals in 95 human cell lines. (DOCX) [file pgen.1010599.s016.docx]

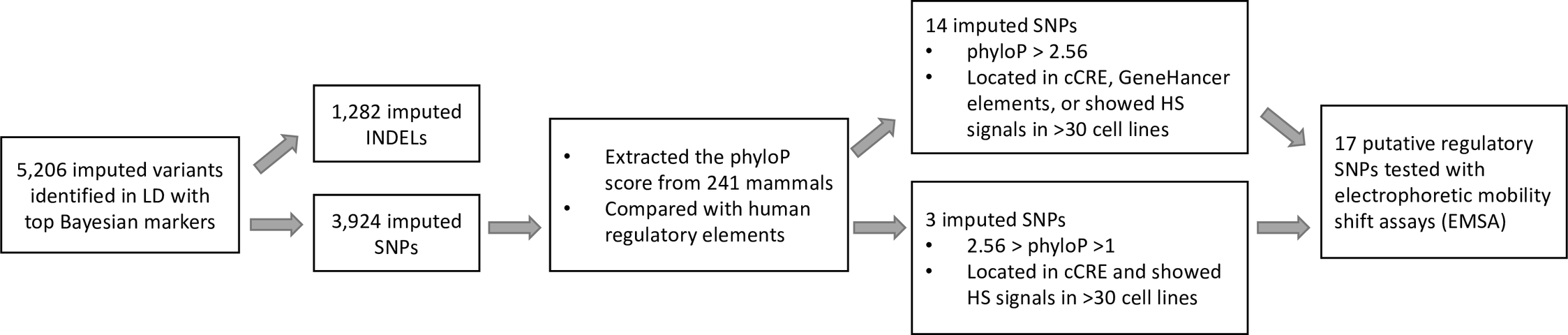


**S2 Fig. Flow chart of selection of 17 putative regulatory SNPs.** Imputed SNPs in LD with top markers from Bayesian analysis were compared with the candidate Cis-Regulatory Elements (cCRE, ENCODE), promotor and enhancer elements (GeneHancer), and Hypersensitivity (HS) signals in 95 human cell lines.
