# Supplementary material for: Bayesian mixed model analysis uncovered 21 risk loci for chronic kidney disease in boxer dogs
Source: PLoS Genet. 2023 Jan 24;19(1):e1010599. doi: 10.1371/journal.pgen.1010599 (PMC9897549; doi:10.1371/journal.pgen.1010599)
Supplement: S3 Fig — (A) A 569 Kb CKD region was found on chr28. It contains one candidate marker (green dot) from Bayesian analysis. (B) A putative regulatory SNP (C14) was found in the intergenic region in the CKD region. EMSA confirmed the allele-specific binding of C14 in both HEK293 and MDCK cell lines. (DOCX) [file pgen.1010599.s017.docx]

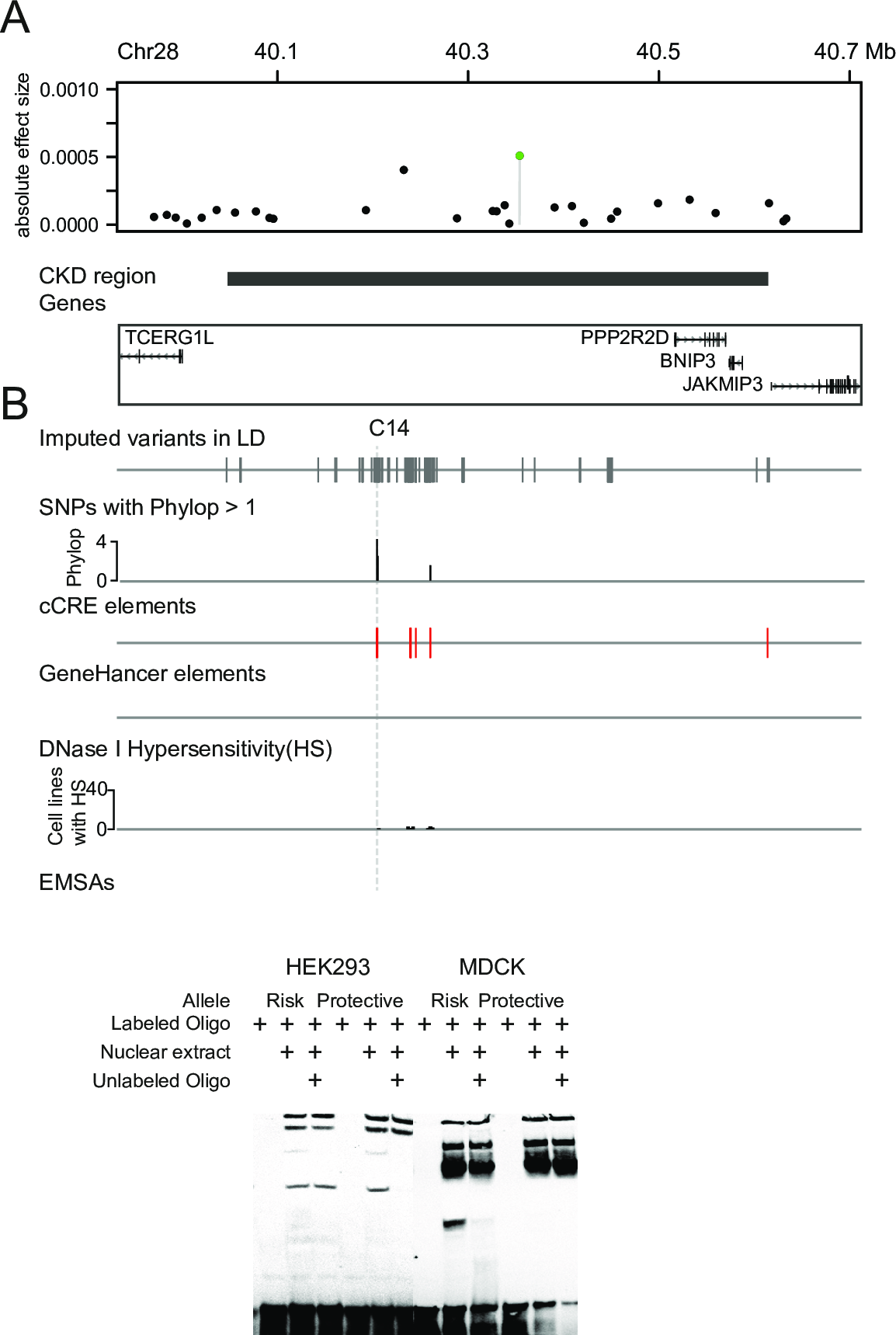


**S3 Fig. Chronic kidney disease (CKD) region on chr28 (chr28:40-40.6 Mb).** (**A**) A 569 Kb CKD region was found on chromosome28. It contains one candidate marker (green dot) from Bayesian analysis. (**B**) A putative regulatory SNP (C14) was found in the intergenic region in the CKD region. EMSA confirmed the allele-specific binding of C14 in both HEK293 and MDCK cell lines.
