# Supplementary material for: Bayesian mixed model analysis uncovered 21 risk loci for chronic kidney disease in boxer dogs
Source: PLoS Genet. 2023 Jan 24;19(1):e1010599. doi: 10.1371/journal.pgen.1010599 (PMC9897549; doi:10.1371/journal.pgen.1010599)
Supplement: S4 Fig — The first two components explained 15.5% and 6.5% genetic variation, respectively. In general, boxers with different origins were mixed as one cluster, with controls and cases evenly distributed. (DOCX) [file pgen.1010599.s018.docx]

**
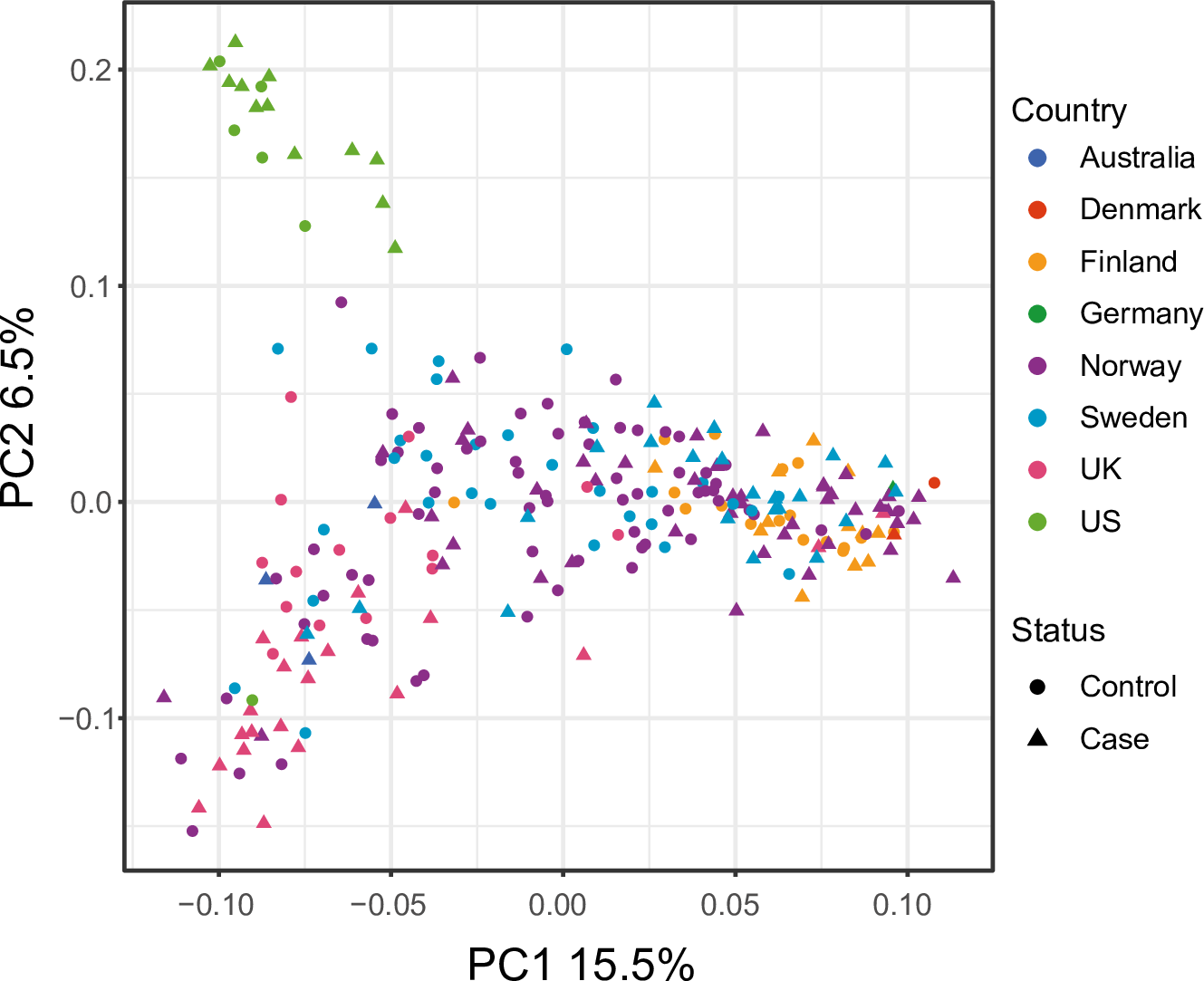
**

**S4 Fig. Principal component analysis (PCA) plot showed the population structure of 254 boxers**. The first two components explained 15.5% and 6.5% genetic variation, respectively. In general, boxers with different origins were mixed as one cluster, with controls and cases evenly distributed.
